# Oxygen-dependent biofilm dynamics in leaf decay: an in vitro analysis

**DOI:** 10.1038/s41598-024-57223-7

**Published:** 2024-03-20

**Authors:** Sabina Karačić, Brianne Palmer, Carole T. Gee, Gabriele Bierbaum

**Affiliations:** 1 Institute of Medical Microbiology, Immunology and Parasitology, University Hospital Bonn, Venusberg‐Campus 1, 53127 Bonn, Germany; 2 Bonn Institute of Organismic Biology, Division of Paleontology, University of Bonn, 53115 Bonn, Germany

**Keywords:** Biofilms, Microbiology, Plant sciences

## Abstract

Biofilms are important in the natural process of plant tissue degradation. However, fundamental knowledge of biofilm community structure and succession on decaying leaves under different oxygen conditions is limited. Here, we used 16S rRNA and ITS gene amplicon sequencing to investigate the composition, temporal dynamics, and community assembly processes of bacterial and fungal biofilms on decaying leaves in vitro. Leaves harvested from three plant species were immersed in lake water under aerobic and anaerobic conditions in vitro for three weeks. Biofilm-covered leaf samples were collected weekly and investigated by scanning electron microscopy. The results showed that community composition differed significantly between biofilm samples under aerobic and anaerobic conditions, though not among plant species. Over three weeks, a clear compositional shift of the bacterial and fungal biofilm communities was observed. The alpha diversity of prokaryotes increased over time in aerobic assays and decreased under anaerobic conditions. Oxygen availability and incubation time were found to be primary factors influencing the microbial diversity of biofilms on different decaying plant species in vitro. Null models suggest that stochastic processes governed the assembly of biofilm communities of decaying leaves in vitro in the early stages of biofilm formation and were further shaped by niche-associated factors.

## Introduction

Microbial biofilms are ubiquitous in natural environments^1^. The microbial interactions within biofilm communities are complex, and the sessile microbial lifestyle of biofilms provides many advantages to its members, such as increased nutrient availability, protection from dehydration, increased tolerance to stressors, and facilitated gene transfer^2–4^. Leaves, as the dominant above-ground plant structures, provide a habitat for numerous microorganisms^5,6^. On leaf surfaces, bacteria are the most prevalent colonizers, followed by filamentous fungi and yeasts and initial microbial activity plays a key role in the decomposition of soft tissues^7^.

Numerous studies of plant microbial communities have focused on microbial composition and plant–microbiome interactions in the rhizosphere, seed and sprouts, endosphere, and phyllosphere^8–10^. While the role of bacteria in leaf colonization and biofilm formation has been investigated, information on leaf biofilm-associated community structure and dynamics on decaying leaves is limited. Knowledge about plant leaf biofilm formation and development is mainly based on defined aerobic cultures of plant-pathogenic bacteria using fresh fruits and vegetables as host plants^11^. Such studies cannot explain the complexity of biofilm structure and dynamics on decaying plant leaves in natural settings because natural ecosystems contain a high diversity of unculturable microorganisms with complex interactions and functions Moreover, the mechanisms determining the composition and development of the biofilm communities on leaves are still poorly understood.

Studies on leaf microorganisms have emphasized the importance of biofilms in the process of plant tissue degradation^1^. However, previous research showed that microbial biofilms can also have a protective role if the degradative microbial activity is slowed down, for example, in the presence of heavy metals which are toxic to bacteria. In this case, the anionic charges and high metal ion-binding capacity of biofilms serve as nucleation points for mineral precipitation and potential preservation^7,12^. In addition, the fate of soft tissues has often been associated with various oxygen conditions, which will change bacterial metabolism, thereby influencing the pH and chemistry of the immediate surroundings^13–16^. It is still a matter of debate which conditions favour decay and which favour preservation, and this may also depend on the chemistry of the surrounding medium. However, even our basic understanding of how bacteria and fungi form biofilms on decaying leaves under differing conditions is limited, for example, under aerobic and anaerobic conditions.

Our study aimed to observe the development of biofilms on leaves decaying in vitro and investigate the effect of oxygen conditions and time on biofilm microbial community structure.

Deciduous and evergreen leaves harvested from three terrestrial plant species were immersed in lake water under laboratory conditions to characterize microbial biofilm community composition and assembly during the decay process under aerobic or anaerobic conditions. To identify the leaf biofilm microbiota at specific stages of decay, we examined the temporal dynamics of the microbial community over 3 weeks. In addition, we looked for bacteria and fungi that might be involved in early mineralization.

High throughput amplicon sequencing of 16S rRNA and ITS genes was used to assess the composition, diversity, and community assembly of bacterial and fungal biofilms on the decaying leaves. To our knowledge, this is the first study to examine the temporal dynamics and structure of microbial biofilm communities of plant leaves during decay under aerobic and anaerobic conditions.

## Results

### *Biofilm growth on the leaf surfaces *in vitro

**Figure 1:**
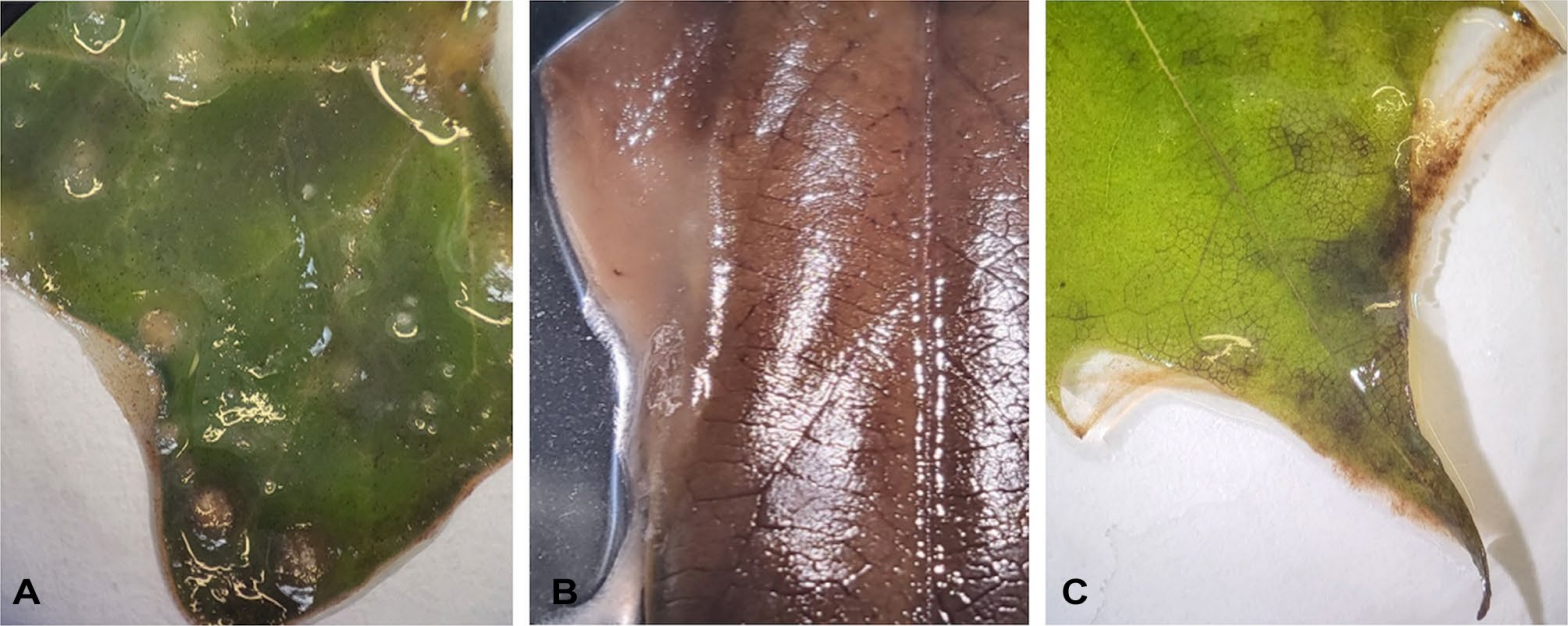
Biofilms developed on decaying leaves in vitro after three weeks of incubation under aerobic conditions: (**A**) *Hedera helix* (**B**) *Lonicera henry*i (**C**) *Acer sp.*

After one week of exposure to lake freshwater, very thin biofilms were observed on all leaves. However, after two weeks, a thick, slimy layer was formed on leaf surfaces under aerobic conditions. In contrast, leaves under anaerobic conditions were consistently covered with a thin biofilm layer. Although all leaf samples showed signs of decay after two weeks of incubation (Fig. 1), there were no visible differences in the appearance and thickness of biofilms between different species under either oxygen conditions. SEM images show an increased presence of bacteria and fungi on the leaf surfaces under both oxygen conditions after three weeks (Supplementary Figs. 1–3).

### Bacterial biofilm communities

**Figure 2:**
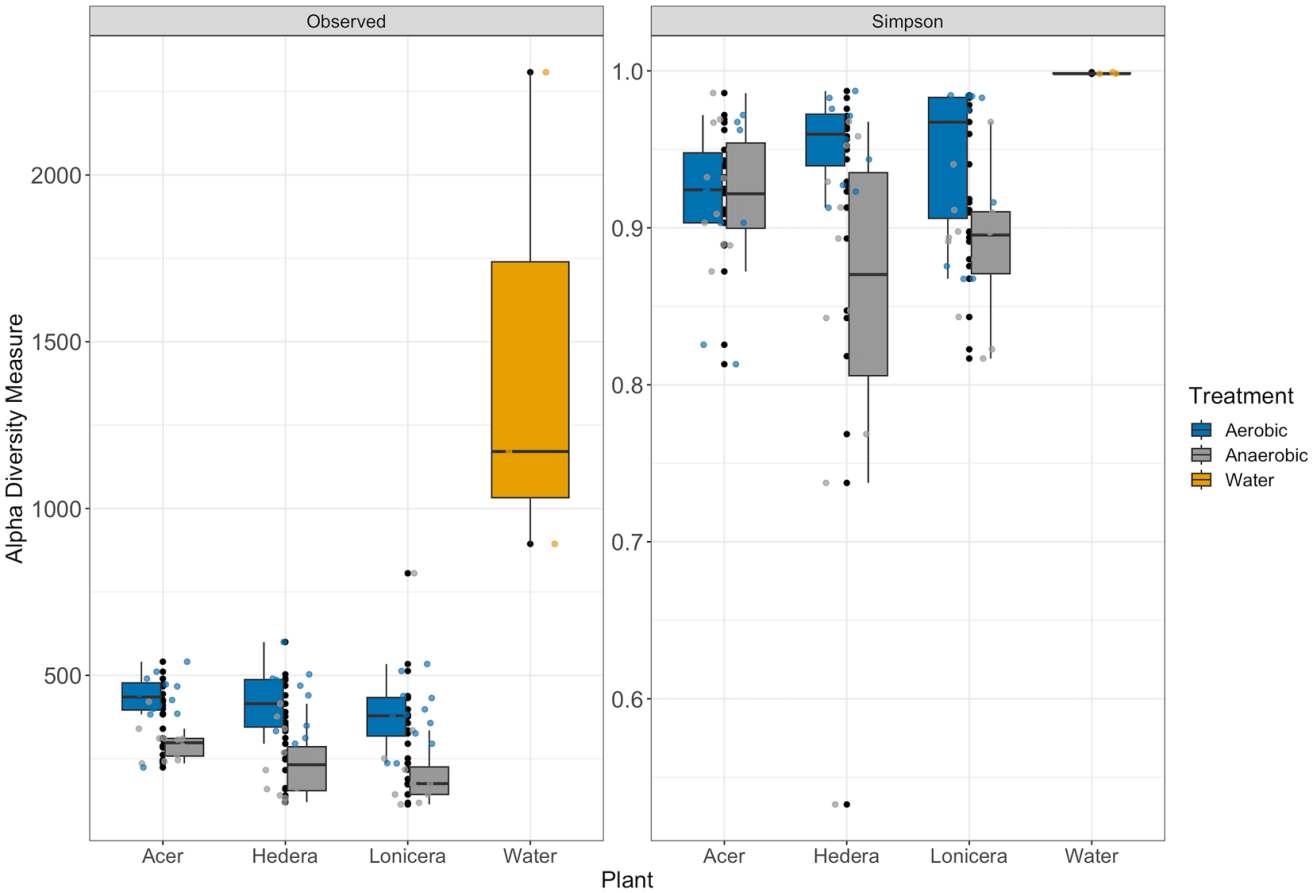
Alpha diversity of leaf prokaryotic biofilm communities over time and water samples. The boxes show median, 25, and 75 percentiles. The vertical lines show minimum values excluding outliners, and the dots show outliners. The colours represent the oxygen conditions and water.

Taxonomic richness was measured as alpha diversity (observed number of ASVs) and Simpson’s evenness for biofilm communities under both oxygen conditions (Fig. 2). Alpha diversity of bacterial biofilm communities increased over time (ANOVA, p < 0.001) in the aerobic assay, and decreased under anaerobic conditions. Differences in prokaryotic richness between aerobic and anaerobic conditions were statistically significant (ANOVA, p < 0.021) with the higher observed richness of samples in aerobic conditions. Conversely, the evenness was similar between both oxygen conditions.

A larger fraction of the ASVs were unique to the leaf biofilms during aerobic incubation (1822 ASVs, 42% of ASVs) compared to anaerobic incubation (1143 ASVs, 26.4% of ASVs). In total, 31.6% of ASVs were shared between all samples (Supplementary Fig. S4A).

The alpha diversities of prokaryotic biofilm communities from the three different plants were not statistically different and they shared 20.1% of all ASVs (Fig. S4B). Biofilms from *Acer* leaves had the highest percentage of exclusive ASVs (1043 ASVs, 23.2%) compared to *Lonicera* (966 ASVs, 21.5%) and *Hedera* (880 ASVs, 19.5%) plants. Interestingly, the richness of the total biofilm plant communities was much lower than the diversity of planktonic bacteria in the water (Fig. 2 & Supplementary Fig. S5).

**Figure 3:**
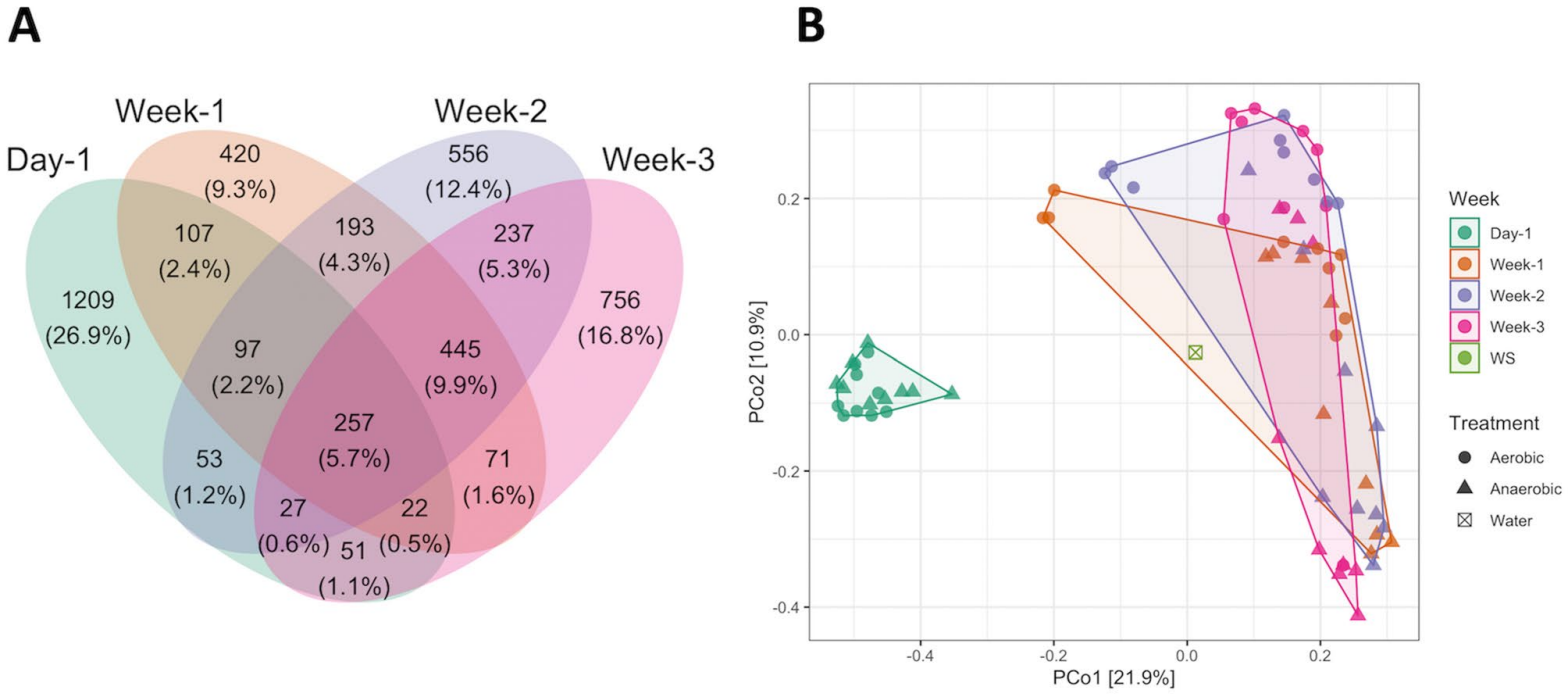
(**A**) Venn diagram showing the shift of the bacterial communities in 72 biofilm samples over time, day 1, green; week 1, orange; week 2, violet; week 3, magenta. (**B**) Ordination by PCoA of bacterial community data based on Bray–Curtis dissimilarity; WS, lake water sampled on day 1.

In total, 1209 ASVs (26.9%) were unique to all plant-biofilm samples at the beginning of the experiment, while 756 ASVs (16.8%) were exclusive to microbial communities sampled after three weeks of study (Fig. 3A). The Venn diagram shows that microbial communities changed over time with a consistent core number of community members (257 ASVs, 5.7%) which were present during the whole experiment (Fig. 3A).

Unconstrained ordination of the prokaryotic biofilms (Bray–Curtis distance-based PCoA- Principal Correspondence Analysis) revealed that the bacterial communities were structured by the oxygen conditions (Supplementary Fig. S6A). The PERMANOVA analysis (p = 0.021) confirmed that leaf biofilms were significantly different under aerobic and anaerobic conditions (Table S1). In addition, leaf biofilm communities varied significantly compared to the planktonic microorganisms in the fresh lake water that had been used as inoculum and medium in the experiment (Supplementary Fig. S6A).

The changes in bacterial biofilm community over time (Fig. 3B) indicate that all biofilm samples followed the same temporal trajectory, although there was no significant difference in microbial biofilm communities between the plants (p = 0.235).

**Figure 4:**
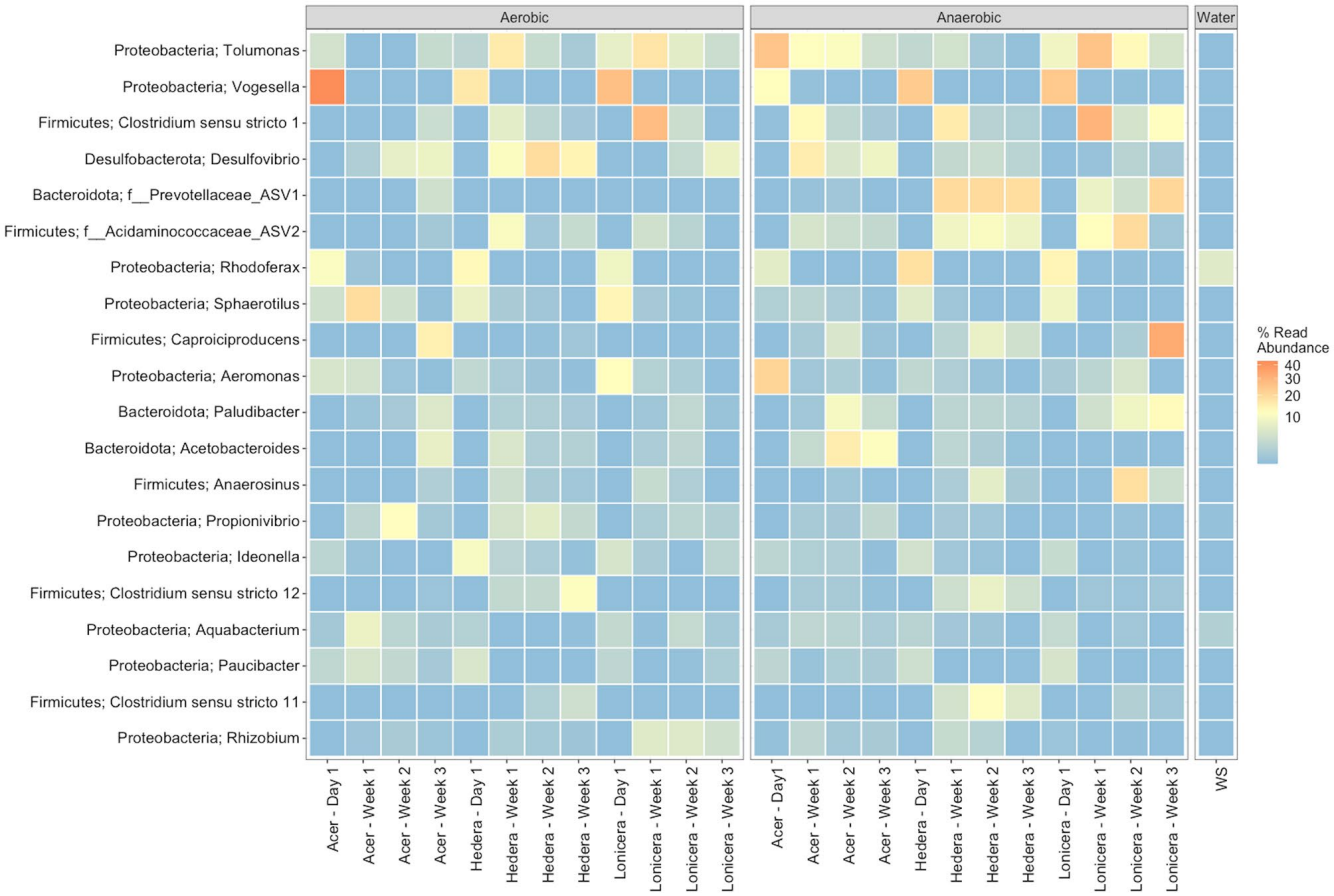
Heatmap of the 20 most abundant bacterial genera or ASVs in the leaf biofilms. The numeric values indicate the percent relative abundance in the sample which are colour-coded (orange indicates high relative abundance and blue indicates low relative abundance). Triplicate biofilm and water samples were merged.

Although differences in the biofilm community structures between treatments and during the experiment were evident, the microbial communities contained many shared taxa (Fig. 4).

Taxonomic analysis of the 16S rRNA ASVs showed that Proteobacteria was the dominant phylum, representing 50% of the total prokaryotic biofilm communities (Fig. 4, Supplementary Fig. S7) followed by Firmicutes, Bacteroidota, Desulfobacterota, Verrucomicrobiota, and Actinobacteriota. In addition, Acidobacteriota, Cyanobacteria, and Patescibacteria were present with relative abundances > 1.5% of the total bacterial communities in the 72 biofilm samples (Supplementary Fig. S7).

The genera *Vogesella, Tolumonas, Rhodoferax, Sphaerotilus,* and *Aeromonas* decreased in relative abundance over time under aerobic and anaerobic conditions, whilst *Clostridium, Desulfovibrio,* and *Caproiciproducens* increased (Fig. 4, Supplementary Fig. S7). Furthermore, ASV1 within Prevotellaceae, and ASV2 assigned to Acidaminococcaceae, *Paludibacter*, *Anaerosius,* and *Rhizobium* increased in abundance over time. Some orders, most notably *Bacteroidales*, *Ocillospirales*, *Acidaminococcales*, and *Clostridiales* were more abundant in anaerobic conditions. Overall, only 2 ASVs within *Rhodoferax* and *Aquabacterium* were observed in high abundance (> 1%) in the plant biofilms and water samples with a decreasing trend over time. The exception is *Aquabacterium* which increased abundance over experimental time in the *Acer* biofilm samples.

**Figure 5:**
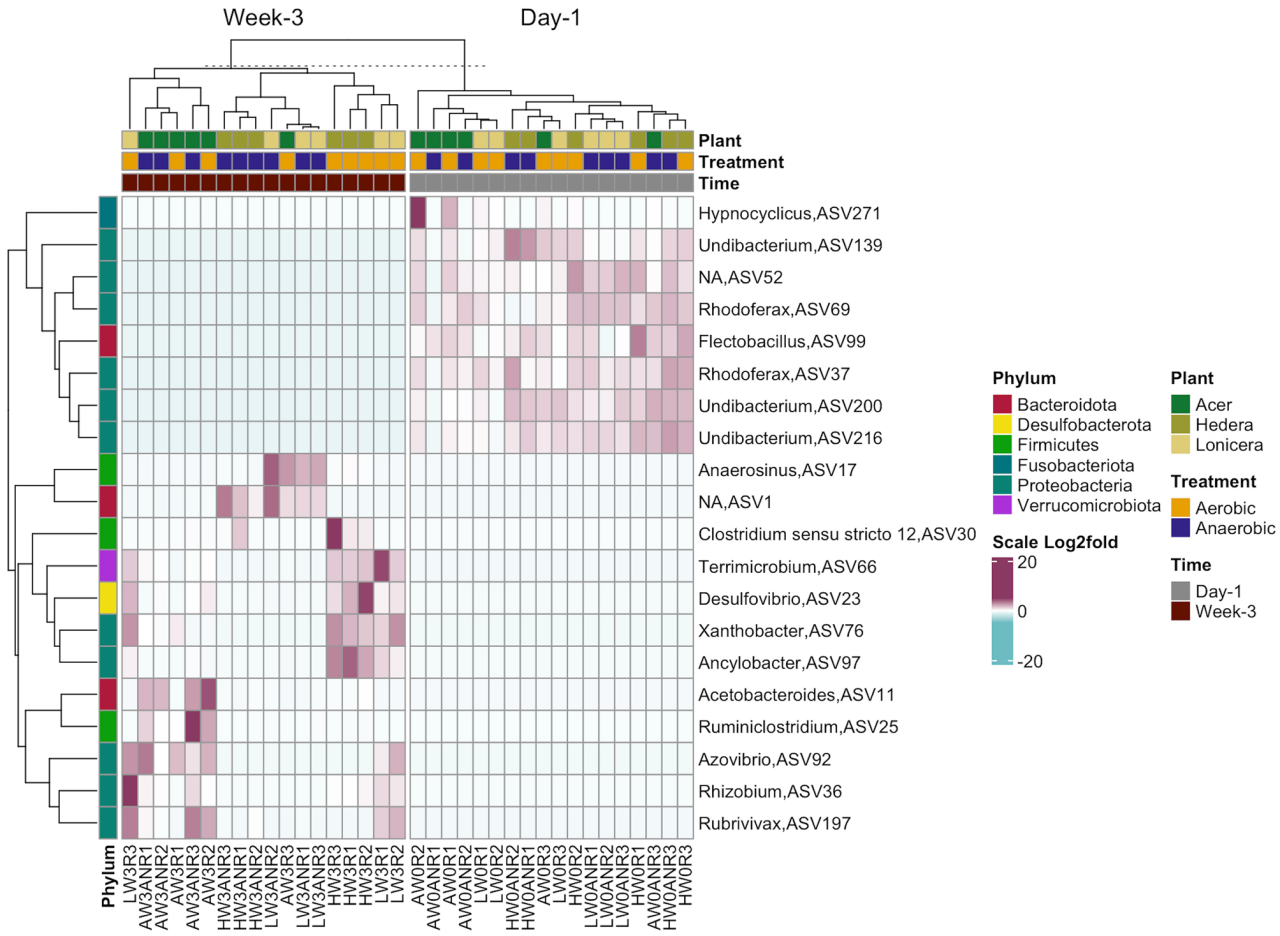
Heatmap showing significantly different amplicon sequence variants (ASVs) abundant across time (day 1—week 3) in leaf biofilms samples based on DESeq2 results (p_adj._ < 0.01). Samples have been grouped initially by time (day 1, week 3), then by plant genus (*Acer*, *Hedera*, *Lonicera*) and treatment (aerobic, anaerobic). ASVs are labelled according to phylum level taxonomy indicated on the left. The shaded cells represent the relative abundance of each ASV on a Log2fold scale with dark red indicating higher abundance and blue indicating lower abundance.

To investigate how time and oxygen conditions affected the abundance of individual ASVs in leaf biofilms, we used DESeq2 which determines ASVs with statistically significant differences (p < 0.01) in abundance over time (Fig. 5).

Several ASVs within *Undibacterium* and *Rhodoferax* were prevalent in the plant biofilms at the beginning of the experiment. In addition, *Hypnocyclicus* and *Flectobacillus* ASVs were highly abundant in many biofilm samples and decreased with time. After three weeks of the experiment, leaf biofilms in vitro were enriched with *Anaerosius*, *Clostridium*, *Terrimicrobium*, *Desulfovibrio*, *Ruminiclostridium*, *Rhizobium*, *Acetobacteroides and Azovibrio*. Comparing the different oxygen conditions, several ASVs classified as *Paludibacter*, *Caproiciproducens,* and *Clostridium *sensu stricto* 1* were significantly more abundant under both oxygen conditions (Fig. S8).

### Fungal biofilm communities

**Figure 6:**
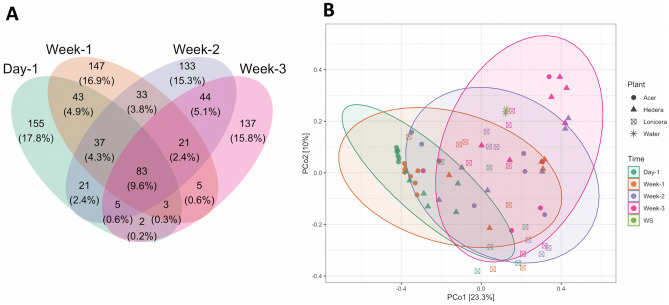
(**A**) Venn diagrams of the fungal communities over time. (**B**) Ordination by PCoA of fungal biofilm data based on Bray–Curtis dissimilarity.

Fungal communities shared 147 ASVs (16.9%) among different plant species with the highest number of unique ASVs (32.2%, 280 reads) belonging to the *Lonicera* biofilm samples. Moreover, 31.9% (277 ASVs) were shared between all leaf biofilm samples from both experimental treatments, but only 83 ASVs (9.6%) were present during the entire duration of the experiment in biofilms on all leaves (Fig. 6A, Supplementary Fig. S9).

Beta diversity PCoA plots revealed that fungal biofilm communities did not differ between oxygen treatments (Supplementary Fig. S6B) or between different plants. However, the fungal community changed over time in a Bray–Curtis distance matrix (Fig. 6B).

**Figure 7:**
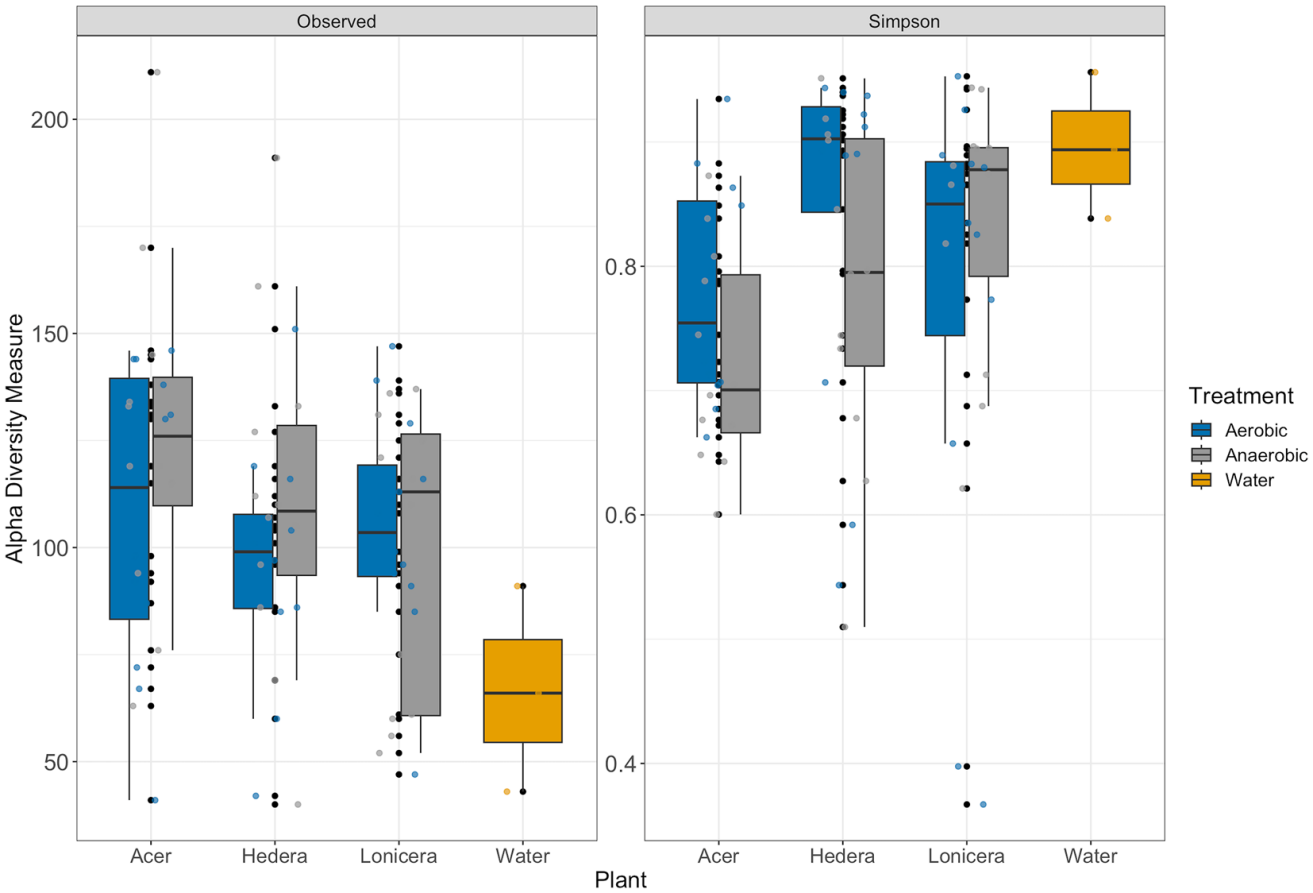
Alpha diversity of leaf fungal biofilm communities from different plants over time. The boxes show median, 25, and 75 percentiles. The vertical lines show minimum values excluding outliners, and the dots show outliners. The colours represent the oxygen conditions and water.

The alpha diversity of leaf biofilms in vitro showed significant changes in the fungal community over time. Fungal richness decreased during the aerobic incubation (ANOVA, p < 0.002), whereas it remained relatively stable under anaerobic conditions during the three weeks of the experiment (Fig. 7). In addition, the low fungal biofilm diversity was confirmed by an average Simpson index lower than 0.9. The Simpson indices for fungal leaf biofilms of different plants were *Hedera* > *Lonicera* > *Acer* (Supplementary Fig. S5B). Fungal richness was lower in the water samples than in the leaf biofilms.

**Figure 8:**
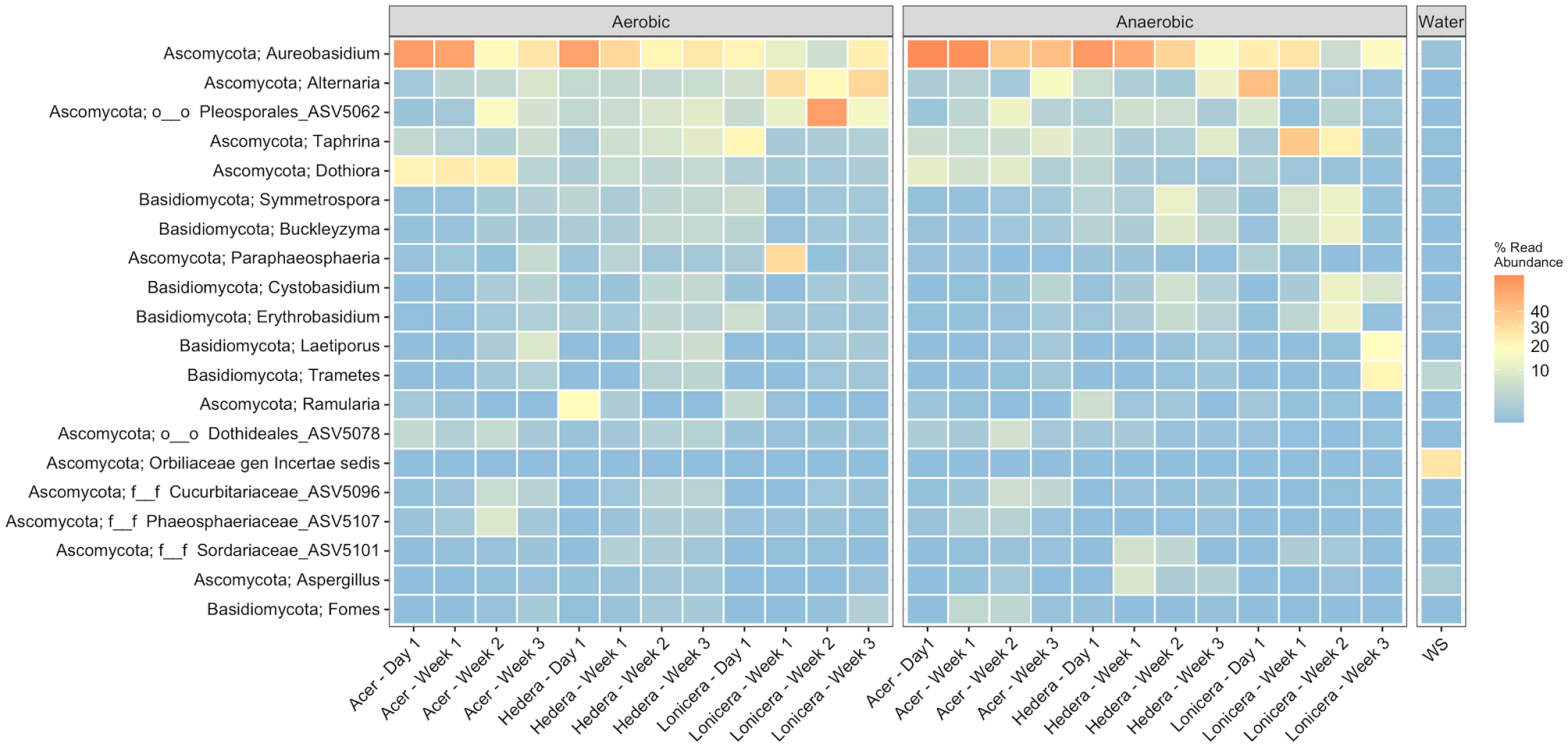
Heatmap of the top 20 most abundant fungal ASVs in the leaf biofilms in vitro during a three-week experiment. Triplicate biofilm and water samples were merged. The colour bar indicates the percent relative abundance in the sample.

Ascomycota was the dominant phylum in leaf biofilms in both treatments with a relative abundance of 78.51%, followed by Basidiomycota (10.43%). At the genus level, a high abundance of *Aureobasidium*, *Alternaria*, *Taphrina,* and *Dothiora* was observed in all leaf biofilm samples. Furthermore, the ASV5062 within Pleosporales and one unclassified ASV (phylum incertae sedis) were detected in high abundance across all leaf biofilms (Fig. 8).

**Figure 9:**
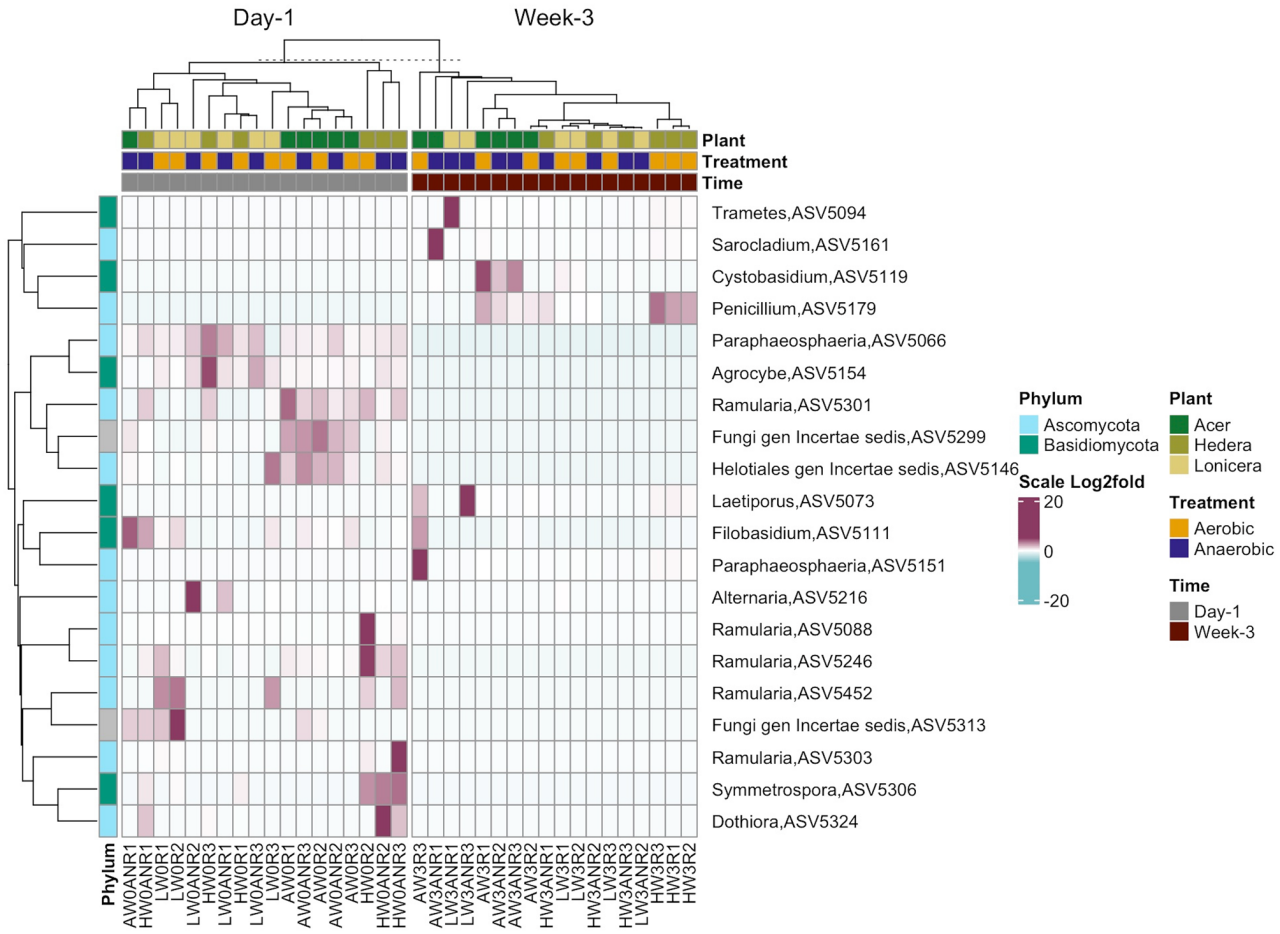
Heatmap showing significantly different fungal ASVs abundant across time (week 0- week 3) based on DESeq2 results (p_adj_ < 0.01). Samples have been grouped initially by time (week 0, week 3), then by plant genus (*Acer*, *Hedera*, *Lonicera*) and treatment (aerobic, anaerobic). ASVs are labelled according to phylum level taxonomy indicated on the left.

Interestingly, the abundance of ASVs assigned to *Laetiporus* and *Trametes* increased in leaf biofilms over time in both oxygen treatments. According to DEseq2 (Fig. 9), *Trametes*, *Sarocladium*, *Laetiporus*, *Cystobasidium,* and *Penicillium* were enriched in the leaf biofilms after three weeks of the experiment. Furthermore, ASVs affiliated with the major plant pathogenic fungi *Alternaria* and *Ramularia* were dominant in the leaf biofilms at the beginning of the experiment. *Alternaria* increased in abundance over time except for *Lonicera* biofilms from anaerobic assays. In contrast, five ASVs assigned to the fungus *Ramularia* decreased in abundance over time.

### Microbial community assembly

**Figure 10:**
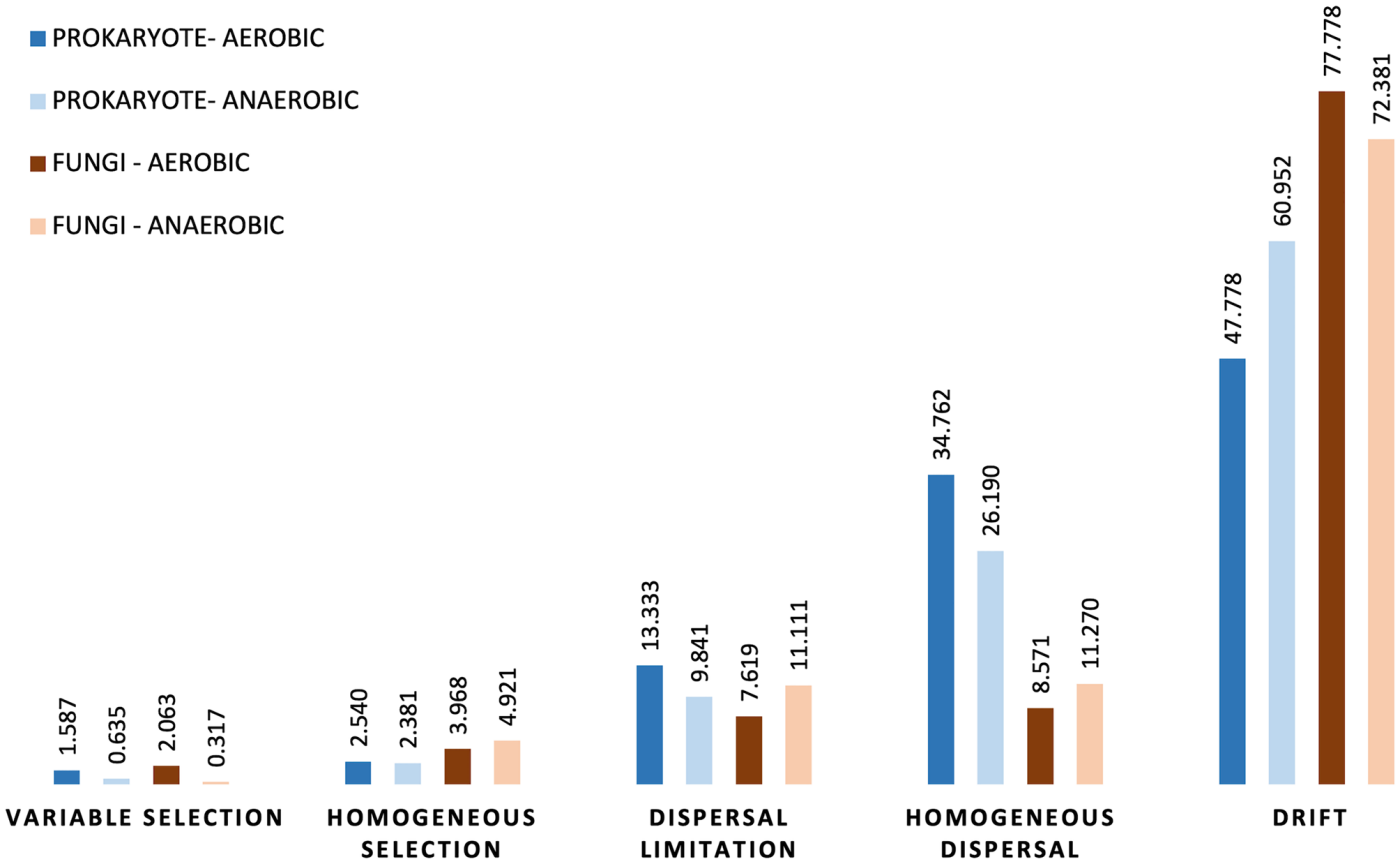
Plots showing the relative contribution (%) of various ecological assembly processes governing the turnover of bacterial and fungal leaf biofilm communities.

Our results showed that stochastic processes (β-NTI < 2, ANOVA, p < 0.01, Fig. S10) determine the assembly of leaf biofilm communities. The null model of betaNRI (Net Relatedness Index) revealed temporal phylogenetic turnover in bacterial and fungal leaf biofilm communities (Supplementary Fig. S10). To understand which stochastic processes dominate the assembly of leaf bacterial and fungal biofilm communities, we used Bray–Curtis-based Raup-Crick calculations within the 'microeco’ package. The results showed that drift was the dominant ecological process driving the colonization of the leaves on day 1 by prokaryotic and fungal communities (Fig. 10, Supplementary Table S2). Later the deterministic factors shaped the formation of the bacterial leaf biofilms (47.78–60.95%) and fungal biofilm communities (72.38–77.77%). Moreover, homogeneous dispersal (26.19–34.47%) and dispersal limitations (9.84–13.33%) contributed to the bacterial biofilm community succession. In addition, deterministic processes like variable and homogeneous selection influenced bacterial and fungi leaf biofilm assembly. However, normalized stochasticity ratio (NRT) and standard effect size (SES) results indicate that deterministic factors were dominant in mature biofilms after one week of the experiment. Interestingly, the relative contribution of niche-based deterministic processes (variable and homogeneous selection) in the assembly of leaf biofilms was higher for fungal communities compared to prokaryotes.

## Discussion

In this study, we detailed differences in the successional dynamics of the bacterial and fungal biofilm communities in aerobic and anaerobic in vitro environments. The results of this study show that the availability of oxygen and incubation time were the main factors influencing the microbial diversity of biofilms on different decaying plant species. The type of leaf, whether evergreen or deciduous and its biological affinity did not play an important role.

### Changes in microbial richness and diversity during biofilm formation

The biofilm communities on the leaves were distinctly different from the planktonic community in the fresh water at the beginning of the experiment with a significantly higher abundance of bacterial and fungal taxa which were not observed in the water. This suggests that members of the leaf-colonizing microbiota were early biofilm-forming microorganisms that established a primary biofilm environment for the subsequent colonizers. However, it should be noted that plant leaves had been incubated for 24 h before the first sampling, it is possible that a certain number of microorganisms from the water colonized the surface of the leaves, but they were not present in high abundance. Interestingly, the bacterial richness and diversity of leaf biofilms in our study were lower than the bacterial richness in the water. This result agrees with previous studies where bacterial community diversity of plant phyllosphere samples was lower than in soil, marine environments, or, the rhizosphere^17^. However, the microbiome of harvested leaves used in this experimental study in vitro cannot be compared with the microbiome of decaying or fresh leaves in nature due to the closed environment of the study.

At the beginning of the experiment, the bacterial biofilm community on the leaves of each plant species consisted primarily of Proteobacteria and Firmicutes. Over time, Bacteroidota and Desulfobacterota increased in abundance under both oxygen conditions and it has to be assumed, that the oxygen was partly depleted during the incubation also in the aerobic experiment and especially in the lower layers of the biofilms. This result supports previous studies where Proteobacteria predominate the phyllosphere of distinct plant species^18–20^ and Firmicutes and Bacteriodota increased during the decomposition of the macrophyte leaf litter^20^. Interestingly, the alpha diversities of prokaryotic and fungal biofilm communities from the three different plants were not statistically different.

We observed a significant increase in bacterial alpha diversity richness over time and a decrease in diversity for the fungal biofilm members. Various studies showed an increase in alpha diversity and abundance over time under aerobic conditions that follows patterns of species-time relationships observed in bacterial communities of phyllosphere and many other microbial ecosystems^19,21,22^. As the biofilm matures, bacteria and fungi start to metabolize the cell polymers and excrete metabolites creating more environmental niches and causing changes in the biofilm community^23^.

### Biofilm development and succession of bacterial communities

In this study, species of the genera *Tolumonas*, *Aeromonas*, *Vogesella*, *Rhodoferax*, *Sphaerotilus*, *Idionella*, *Paucibacter*, *Aquabacterium*, *Undibacterium,* and *Flectobacillus* were potential biofilm formers. The genus *Aeromonas* is a strong decomposer of soft tissues as well as a biofilm former^24^. These facultatively anaerobic bacteria were observed in high abundance during the initial stages of the experiment in all plant biofilm samples. Domination of *Aeromonas* species during initial decay processes has been reported in previous studies about the decomposition of soft tissue materials^14^. In addition, filamentous bacteria of the genus *Sphaerotilus* and motile, microaerophilic species of *Aquabacterium*^25^, capable of accumulating large amounts of slime, were detected in high abundance during the first three weeks of study in the aerobic and anaerobic samples. The sheaths of *Sphaerotilus* may be encrusted with iron and manganese oxides^26–28^.

The ASVs that were assigned to iron-oxidizing bacteria within *Rhodoferax* and *Undibacter* were enriched exclusively on the first sampling occasion, suggesting that a lack of iron ions in the incubation medium caused a microbial shift within leaf biofilms in vitro. Over time, many ASVs that had been abundant at the beginning of the experiment decreased in abundance while other ASVs increased, thus establishing more diverse biofilm communities.

After one week of incubation when the biofilm layers were well visible, *Clostridium*, *Desulfovibrio*, *Propionivibrio*, Acidaminococcaceae, *Paludibacter,* and *Acetobacteroides* had increased. *Desulfovibrio* is a sulfate-reducing genus that typically grows anaerobically, but certain strains tolerate the presence of oxygen. Interestingly, in an anoxic environment, *Desulfovibrio* can oxidize organic matter and generate hydrogen sulfide as a final product, which may react with iron and lead to the formation of iron sulfide and further formation of pyrite^29,30^. From fossilization studies, it is known that pyritization is important for the preservation of plant tissues^16,31,32^.

Although *Paludibacter* and *Clostridia* were enriched under both oxygen conditions, fermentative bacteria were dominant in anaerobic samples. The genus *Clostridium* is widely distributed in soils and plants, but only a few studies have shown that diverse strains can colonize plants^33^. In our experiment, several clusters of *Clostridium* were observed in the biofilms including *Clostridium *sensu stricto* 1*, *Clostridium *sensu stricto 11, and *Clostridium *sensu stricto* 12*. Sarkar et al. (2022) emphasize that clostridia are effective H_2_ and acid producers using carbohydrates such as cellulose and sucrose as substrates^34^. Numerous studies showed that saprophytic clostridia are associated with the decay of plants, phytoplankton, animal and human tissues^14,35^.

The genus *Acetobacteroides* also increased in the *Acer* leaf biofilm samples over time. These carbohydrate-fermenting bacteria produce acetate by degrading butyrate and propionate comparable to some clostridia. Previous studies showed that acetobacteria are involved in plant and animal decay processes when an anaerobic environment has been established within the biofilm or in the environment^14,36^. Moreover, ASVs belonging to the fermentative family *Prevotellaceae* and the genus *Caproiciproducens* were observed in higher abundance in the anaerobic incubation in the later stages of the experiment. *Prevotella* is associated with glycan degradation^37^, whilst members of the genus *Caproiciproducens* can produce acetic acid, butyric acid, caproic acid, lactic acid, and H_2_ as metabolic products^38,39^. In addition, ASVs assigned to *Acidaminococcaceae,* which use numerous amino acids as their carbon sources, were detected in the leaf biofilms from anaerobic assays. After three weeks, nitrogen-fixing bacteria *Rhizobium* and *Xanthobacter* were enriched in the leaf biofilm samples (Fig. S11). Both genera can produce large amounts of EPS and enhance the formation of calcium carbonate^40,41^.

### Biofilm development and succession of fungal communities

Fungi have major roles in the decomposition of organic matter and plants^20,42^. Numerous studies showed that they are involved in mineral and metal deposition, transformations, and biomineralization^43–46^. In this study, fungi detected in the leaf biofilms were mainly saprotrophs and pathotrophs (Supplementary Fig. S12).

A PCoA plot based on the Bray–Curtis dissimilarity (Fig. 6B) and betaNRI plots (Supplementary Fig. S10) revealed a succession of fungal communities with the highest betaNRI in biofilms sampled after three weeks of the experiment. We observed that fungal richness decreased over time with higher Simpson´s evenness in the aerobic incubation. Therefore, we can assume that time was the main factor shaping fungal biofilm communities. The fungi *Laetiporus* and *Trametes* exert a strong antimicrobial effect^47^ and were enriched in the biofilms at the later stages of the experiment. Members of both genera are wood-decaying fungi that decompose cellulose, hemicellulose, and lignin^48^. Furthermore, *Trametes* strains secrete extracellular fatty acid droplets^49^ and corrode apatite under glucose-limited conditions^50^. Graz et al. 2023 confirmed the oxalic acid-degrading activity of *Trametes* and *Laetiporus*^51^*.* Oxalic acid is commonly secreted by the majority of fungi during the wood decay process, and it is linked with the ability to chelate metals^51,52^. The endophytic fungi *Sarocladium* and *Penicillium* were enriched in the biofilms according to Deseq2. These fungi are common plant pathogens, but several studies observed enhanced stress tolerance and plant growth-promoting activity^53^. In addition, *Alternaria* and *Aureobasidium* were prevalent in all leaf biofilm samples during the experiment. *Aureobasidium* is a ubiquitous yeast saprotroph that can promote plant growth^54^ and produces volatile organic compounds with antifungal properties against *Alternaria*^55^. Interestingly, Hou et al. 2011 observed precipitations of calcium carbonate by *Alternaria* during nitrate removal from the medium^56^.

### Ecological processes governing the leaf biofilm assembly

The betaNRI values of biofilm communities under both oxygen conditions at the beginning of the experiment were, on average, more phylogenetically dispersed or random, and, after one week of the experiment, biofilm communities were significantly phylogenetically clustered.

In our experimental study in vitro, in addition to the leaf-colonizing flora, the harvested leaves were all inoculated with saprophytes from the lake water used as medium. Therefore, it could be assumed that microbial biofilm communities from different plant species would be phylogenetically clustered over time. In contrast, plant microbial communities in nature often diverge over time^57^. Here, our results showed that stochastic processes dominated the assembly of leaf biofilm communities under aerobic and anaerobic conditions at the beginning of the experiment (Supplementary Fig. S10, Supplementary Table S2). Bray–Curtis-based Raup-Crick calculations confirmed that 77% of turnover in bacterial and 61% of turnover in fungal biofilm communities was assigned to drift. It is well-known that ecological drift is attributed to random processes like immigration or emigration of microbial taxa, and pronounced under conditions where microbial diversity is low^58,59^. A study of bacterial community temporal dynamics confirmed that stochastic processes were important during the colonization of the phyllosphere^60^. However, in our study, the standard effect size of non-random processes on leaf biofilm compositional dissimilarity (Bray–Curtis) was higher than two when compared between plants, between treatments, and between sampling occasions except for the first day of the experiment (Supplementary Table S2). This means that the observed biofilm compositional dissimilarity was higher than expected by a random assembly. Since decaying leaves in our study in vitro were inoculated from the same environmental microbial pool, homogeneous dispersal likely mediated the similarity of leaf biofilm microbial communities. However, we assume that drift was the major process at the beginning of the experiment when plant leaves were inoculated by microorganisms from the lake freshwater, whilst dispersal and selection further shaped biofilm microbial communities over time. Interestingly, several studies showed the same pattern during succession, where the early stages of biofilm formation were influenced by stochastic factors, while selection was dominant in mature biofilms^21,61^.

## Conclusions

This study characterizes microbial community composition and temporal dynamics of decaying leaves under varying oxygen conditions in vitro. Biofilms have a major role in the decay and preservation of soft tissues and therefore understanding underlying microbial community structure and dynamics under different environmental conditions is important. We used harvested leaves to explore the microbial biofilm communities and the mechanisms shaping community assembly processes during decay under aerobic and anaerobic oxygen conditions.

Our results indicate that microbial biofilms were not influenced by plant species, but that oxygen conditions and time were the main factors shaping bacterial and fungal biofilm communities. In succession, bacterial communities increased in alpha diversity and formed distinct biofilms under aerobic and anaerobic conditions. The assembly of biofilm communities of decaying leaves in vitro was primarily governed by stochastic processes at the early stages of biofilm formation and further shaped by niche-based deterministic factors. The presence of decomposer microorganisms during biofilm formation and their succession with other bacteria and fungi capable of fermentation and biomineralization indicated the presence of niches that enable both processes. These results will act as a starting point for further investigations of leaf biofilms within the biomineralization and preservation context.

## Materials and methods

### Sampling and experimental setup

The freshwater used in this study was collected at the Tongrube Lake, Röttgen, Germany (50°40′24.7′′N/7°04′29.6′′E). Tongrube Lake has a natural water inflow and outflow, and it is one of the largest water bodies in Bonn, Germany, with a maximum depth of six meters and an area of 1700 square meters. The lake bottom consists of a clay substrate. Water samples were collected in 1 L sterile glass bottles, then kept at 4 °C during transport to the laboratory. The freshwater was filtered through a sterilized coarse filter (grade 3hw; Binzer & Munktell Filter GmbH, Battenberg, Germany) for the removal of debris, sediment, and larger organisms.

Deciduous leaves of a maple tree, *Acer* sp*.*, and evergreen leaves of ivy, *Hedera helix*, were gathered from plants growing around the lake in Röttgen, while evergreen leaves of Henry’s honeysuckle, *Lonicera henryi*, were collected in a privately owned garden. We collected approximately 30 leaves of each plant species with sterile gloves and placed them into sterilized glass petri dishes. Plant leaf collection complied with relevant institutional, national, and international guidelines and legislation.

Both experimental treatments in this study were conducted at a constant temperature of 24 °C in a laboratory incubator in the dark for three weeks. We immersed 72 leaves in lake water in sterile glass petri dishes. Triplicates were kept together in the same petri dish. Half of the samples were exposed to aerobic conditions, and the other half was placed in anaerobic containers (BD GasPak™ EZ container). The lake water used for the anaerobic setup was treated with N_2_ gas for 20 min and anaerobic conditions were established with a gas generating system, BD GasPak ™ (BD BBL™ CO_2_ gas generators). Sampling in triplicates of each leaf species was performed after 24 h of incubation, then after 7 days, 14 days, and 21 days. Water samples in triplicates were taken directly from the lake on the first day of the study. To document biofilm formation and plant decay, all samples were examined and photographed using a Zeiss Stemi 2000 stereo microscope and a Tescan Vega 4 LMU Scanning Electron Microscope (SEM) after each sampling session.

A total of 72 leaf samples and three water samples were collected for DNA analysis. All samples were manually shaken in 50 ml of 1 × TE buffer and then sonicated in an ultrasound bath (Branson 1210 Ultrasonic Cleaner; Emerson; St. Louis, Missouri, USA) for two minutes to separate the biofilm and microorganisms attached to the leaf surface. Then, planktonic microorganisms from the freshwater samples or detached biofilm microorganisms from the plant samples were collected on PORAFIL NC filters (0.25 µm, Macherey–Nagel GmbH & Co, KG, Germany) using vacuum filtration. The filters were stored at − 20 °C until nucleic acid extraction.

### DNA extraction, amplification, and sequencing

The total genomic DNA of each sample was extracted following the manufacturer’s protocol using a Fast DNA spin kit for soil (MP Biomedicals), which had been found to be optimal for the extraction of DNA from biofilms on decaying leaves^24^.

DNA was eluted in 50 µl Dnase/RNase-free water and the concentration of the extracted nucleic acids was measured using a Qubit 4.0 fluorometer (Thermo Fisher Scientific, USA) with the dsDNA HS assay kit (Invitrogen). The V4 hyper-variable region of the 16S-rRNA gene was amplified in duplicates, using the forward primer 16 s-515F (GTGCCAGCMGCCGCGGTAA) and the reverse primer 16 s-806R (GGACTACVSGGGTATCTAAT) to cover both bacteria and archaea^62,63^. For the determination of the fungal rRNA sequences, the ITS region was amplified using the ITS1F (CTTGGTCATTTAGAGGAAGTAA) and ITS2R (GCTGCGTTCTTCATCGATGC) primers^64^. PCR was conducted using the HotStarTaq Plus Master Mix Kit (QIAGEN) and carried out as follows: initial denaturation at 95 °C for 5 min, denaturation at 95 °C for 30 s, annealing at 53 °C for 40 s, and extension at 72 °C for 1 min for 30–35 cycles, followed by final elongation at 72 °C for 10 min. Paired-end sequencing (bTEFAP) was performed by Molecular research DNA (Shallowater, Texas, USA; http://www.mrdnalab.com) on an Illumina MiSeq sequencing platform (Illumina, San Diego, California, USA) following the manufacturer's instructions^65^. 16S-rRNA and ITS regions of biofilm and water samples were sequenced in triplicate (n = 72 from the leaves, n = 3 from the water).

### Bioinformatics and statistical analysis

The sequence reads were processed using the QIIME2^66^. Sequence quality control, denoising, and chimera filtering were performed using the DADA2 v.1.16^67^ pipeline which determines amplicon sequence variants (ASVs). Taxonomy for the 16S rRNA sequences was assigned using the Silva database v.138^68^, whilst the Unite database was used for the ITS sequences^69^. The dataset was rarefied by subsampling each sample to 46,232 bacterial reads and 1153 fungal reads. ASVs and taxonomy derived from the Qiime2 and DADA2 pipelines for each database were merged using R version 4.2.1^70^. Multivariate statistics, diversity calculations, and data visualization were conducted within the R packages ampvis2 (version 2.7.3)^71^, vegan (version 2.5.1)^72^, phyloseq (version 1.4.0)^73^, picante 1.6.2^74^, ecodist (version 2.0.9)^75^, microeco (version 0.20)^76^, and ggplot2 (version 3.4.2)^77^. Beta diversity was estimated using pairwise Bray–Curtis dissimilarities and visualized using principal coordinate analysis (PCoA). The statistical differences in microbial community compositions were tested using permutational multivariate analysis of variance (PERMANOVA, adonis2 function). Additionally, differences in community richness and evenness were tested by a univariate statistical test Shapiro–Wilk’s followed by a post-hoc Games-Howels test. The DESeq2 method^78^ was used to test the differential abundance of ASVs over time and between different plant species and was carried out in R. Functional assignments of fungal traits at genus levels were determined using the FungalTraits database^79^ within the “microeco” package.

### Estimation of biofilm community assembly processes

Bacterial and fungal leaf biofilm assembly mechanisms conditions were evaluated using the trans_null model and cal_NST functions in “microeco”. The beta net relatedness index (betaNRI) and beta nearest taxon index (betaNTI) were used to determine the relative role of deterministic and stochastic processes. Values between − 2 and + 2 indicate that compositional turnover between samples was governed by stochastic factors, whilst value − 2 or >+ 2 indicates that selection likely dominates assembly processes. To estimate the proportion of inferred ecological processes that shaped biofilm communities on decaying leaves, betaNTI and Bray–Curtis-based Raup–Crick (RCbray) were calculated using null-models (1,000 randomizations). Calculated values represent homogeneous selection (bNTI < -2), heterogeneous selection (betaNTI > 2), dispersal limitation, (|βNTI|< 2 and RCbray >  + 0.95), homogeneous dispersal (|βNTI|< 2 and RCbray < -0.95) and drift ((|βNTI|< 2 and |RCbray|< 0.95) in governing the composition of the leaf biofilm communities.

### Scanning electron microscopy (SEM)

A small piece of every leaf was excised with a sterile scalpel for SEM. These fresh leaf samples were fixed in 70% v/v ethanol + 4% v/v formaldehyde in water for at least 24 h. Later, samples were dehydrated with ethanol and stored in 100% ethanol until critical point drying. Samples were dried in 22 cycles using a Leica EM CPD300 Critical Point Dryer at the Nees Institute for Biodiversity of Plants. Dried samples were coated with a thin layer of palladium with a sputter coater (Cressington Sputter Coater 108 manual, Tesca GmbH, Germany). Coated samples were mounted on carbon tape on individual stubs and investigated under high-vacuum mode using an SE detector at an acceleration voltage of 20 kV in a Tescan Scanning Electron Microscope.

### Supplementary Information

Supplementary Information.

## Data Availability

Raw sequence reads were deposited at the NCBI Sequence Read Archives under the BioProject accession number PRJNA1036544.

## References

[1] Flemming H-C, Wuertz S (2019). Bacteria and archaea on Earth and their abundance in biofilms. Nat. Rev. Microbiol..

[2] O’Toole G, Kaplan HB, Kolter R (2000). Biofilm formation as microbial development. Annu. Rev. Microbiol..

[3] Donlan RM (2002). Biofilms: Microbial life on surfaces. Emerg. Infect. Dis..

[4] Sutherland I (2001). Biofilm exopolysaccharides: A strong and sticky framework. Microbiology (Reading).

[5] Bashir I (2022). Phyllosphere microbiome: Diversity and functions. Microbiol. Res..

[6] Lindow SE, Brandl MT (2003). Microbiology of the phyllosphere. Appl. Environ. Microbiol..

[7] Dunn KA, McLean RJC, Upchurch GR, Folk RL (1997). Enhancement of leaf fossilization potential by bacterial biofilms. Geology.

[8] Ramey BE, Koutsoudis M, von Bodman SB, Fuqua C (2004). Biofilm formation in plant-microbe associations. Curr. Opin. Microbiol..

[9] Danhorn T, Fuqua C (2007). Biofilm formation by plant-associated bacteria. Annu. Rev. Microbiol..

[10] Bogino PC, Oliva M, Sorroche F, Giordano W (2013). The role of bacterial biofilms and surface components in plant-bacterial associations. Int. J. Mol. Sci..

[11] Chaudhry V (2021). Shaping the leaf microbiota: Plant–microbe–microbe interactions. J. Exp. Bot..

[12] Klymiuk AA (2018). Microbiological Insights into Ecology and Taphonomy of Prehistoric Wetlands.

[13] Hippler D, Hu N, Steiner M, Scholtz G, Franz G (2012). Experimental mineralization of crustacean eggs: New implications for the fossilization of Precambrian-Cambrian embryos. Biogeosciences.

[14] Mähler B (2023). Time-dependent microbial shifts during crayfish decomposition in freshwater and sediment under different environmental conditions. Sci. Rep..

[15] Briggs DEG, Kear AJ (1993). Decay and preservation of polychaetes: Taphonomic thresholds in soft-bodied organisms. Paleobiology.

[16] Janssen K, Mähler B, Mccoy V, Rust J, Bierbaum G (2021). The complex role of microbial metabolic activity in fossilization. Biol. Rev. Camb. Philos. Soc..

[17] Delmotte N (2009). Community proteogenomics reveals insights into the physiology of phyllosphere bacteria. Proc. Natl. Acad. Sci. U. S. A..

[18] Rastogi G (2012). Leaf microbiota in an agroecosystem: spatiotemporal variation in bacterial community composition on field-grown lettuce. ISME J..

[19] Redford AJ, Fierer N (2009). Bacterial succession on the leaf surface: A novel system for studying successional dynamics. Microb. Ecol..

[20] Zhao B, Xing P, Wu QL (2017). Microbes participated in macrophyte leaf litters decomposition in freshwater habitat. FEMS Microbiol. Ecol..

[21] Karačić S, Modin O, Hagelia P, Persson F, Wilén B-M (2022). The effect of time and surface type on the composition of biofilm communities on concrete exposed to seawater. Int. Biodeterior. Biodegrad..

[22] Shade A, Gregory Caporaso J, Handelsman J, Knight R, Fierer N (2013). A meta-analysis of changes in bacterial and archaeal communities with time. ISME J..

[23] Martiny AC, Jørgensen TM, Albrechtsen H-J, Arvin E, Molin S (2003). Long-term succession of structure and diversity of a biofilm formed in a model drinking water distribution system. Appl. Environ. Microbiol..

[24] Janssen K (2021). Elucidating biofilm diversity on water lily leaves through 16S rRNA amplicon analysis: Comparison of four DNA extraction kits. Appl. Plant Sci..

[25] Kalmbach S, Manz W, Wecke J, Szewzyk U (1999). Aquabacterium gen. nov., with description of *Aquabacterium*
*citratiphilum* sp. nov., *Aquabacterium*
*parvum* sp. nov. and *Aquabacterium*
*commune* sp. nov., three in situ dominant bacterial species from the Berlin drinking water system. Int. J. Syst. Evolut. Microbiol..

[26] Schmidt B (2014). Isolation of *Sphaerotilus*-*Leptothrix* strains from iron bacteria communities in Tierra del Fuego wetlands. FEMS Microbiol. Ecol..

[27] Glasauer SM, Burford EP, Gadd GM, Glasauer SM (2022). Transformation of metals and metalloids by microorganisms. Reference Module in Earth Systems and Environmental Sciences.

[28] Seder-Colomina M (2013). Sphaerotilus natans, a neutrophilic iron-related sheath-forming bacterium: Perspectives for metal remediation strategies. Geomicrobiology.

[29] Duverger A (2020). Mechanisms of pyrite formation promoted by sulfate-reducing bacteria in pure culture. Front. Earth Sci..

[30] Kumar SS, Kumar V, Gnaneswar Gude V, Malyan SK, Pugazhendhi A (2020). Alkalinity and salinity favor bioelectricity generation potential of *Clostridium*, *Tetrathiobacter* and *Desulfovibrio* consortium in microbial fuel cells (MFC) treating sulfate-laden wastewater. Bioresour. Technol..

[31] Thiel J, Byrne JM, Kappler A, Schink B, Pester M (2019). Pyrite formation from FeS and H2S is mediated through microbial redox activity. Proc. Natl. Acad. Sci..

[32] Martín-González A, Wierzchos J, Gutiérrez J-C, Alonso J, Ascaso C (2009). Double fossilization in eukaryotic microorganisms from Lower Cretaceous amber. BMC Biol..

[33] Zeiller M (2015). Systemic colonization of clover (*Trifolium repens*) by *Clostridium botulinum* strain 2301. Front. Microbiol..

[34] Sarkar O, Rova U, Christakopoulos P, Matsakas L (2022). Effect of metals on the regulation of acidogenic metabolism enhancing biohydrogen and carboxylic acids production from brewery spent grains: Microbial dynamics and biochemical analysis. Eng. Life Sci..

[35] Mähler B (2022). Adipocere formation in biofilms as a first step in soft tissue preservation. Sci. Rep..

[36] Su X-L (2014). *Acetobacteroides*
*hydrogenigenes* gen. nov., Sp. nov., An anaerobic hydrogen-producing bacterium in the family Rikenellaceae isolated from a reed swamp. Int. J. Syst. Evolut. Microbiol..

[37] Rabee AE, Sayed Alahl AA, Lamara M, Ishaq SL (2022). Fibrolytic rumen bacteria of camel and sheep and their applications in the bioconversion of barley straw to soluble sugars for biofuel production. PLOS ONE.

[38] Choi JY (2023). *Caproiciproducens*
*faecalis* sp. nov., isolated from cow faeces. Curr. Microbiol..

[39] Kim B-C (2015). *Caproiciproducens*
*galactitolivorans* gen. nov., sp. nov., a bacterium capable of producing caproic acid from galactitol, isolated from a wastewater treatment plant. Int. J. Syst. Evol. Microbiol..

[40] Wiegel J, Dworkin M, Falkow S, Rosenberg E, Schleifer K-H, Stackebrandt E (2006). The genus xanthobacter. The Prokaryotes: Volume 5: Proteobacteria: Alpha and Beta Subclasses.

[41] Fraysse N, Couderc F, Poinsot V (2003). Surface polysaccharide involvement in establishing the rhizobium-legume symbiosis. Eur. J. Biochem..

[42] Kubicek CP, Druzhinina IS (2007). Environmental and Microbial Relationships.

[43] Gadd GM, Rhee YJ, Stephenson K, Wei Z (2012). Geomycology: Metals, actinides and biominerals. Environ. Microbiol. Rep..

[44] Brasier MD, Callow RHT, Menon LR, Liu AG, Seckbach J, Oren A (2010). Osmotrophic biofilms: From modern to ancient. Microbial Mats: Modern and Ancient Microorganisms in Stratified Systems.

[45] Gadd GM (2021). Fungal biomineralization. Curr. Biol..

[46] Li Q, Liu D, Jia Z, Csetenyi L, Gadd GM (2016). Fungal biomineralization of manganese as a novel source of electrochemical materials. Curr. Biol..

[47] Figueiredo F (2021). Multi-omics analysis provides insights into lignocellulosic biomass degradation by *Laetiporus sulphureus* ATCC 52600. Biotechnol. Biofuels.

[48] Andlar M (2018). Lignocellulose degradation: An overview of fungi and fungal enzymes involved in lignocellulose degradation. Eng. Life Sci..

[49] Hao G, Barker GC (2022). Fatty acid secretion by the white-rot fungus, *Trametes versicolor*. J. Ind. Microbiol. Biotechnol..

[50] Adeyemi AO, Gadd GM (2005). Fungal degradation of calcium-, lead- and silicon-bearing minerals. Biometals.

[51] Grąz M, Ruminowicz-Stefaniuk M, Jarosz-Wilkołazka A (2022). Oxalic acid degradation in wood-rotting fungi. Searching for a new source of oxalate oxidase. World J. Microbiol. Biotechnol..

[52] Jarosz-Wilkołazka A, Grąz M (2006). Organic acids production by white rot Basidiomycetes in the presence of metallic oxides. Can. J. Microbial..

[53] Potshangbam M, Devi SI, Sahoo D, Strobel GA (2017). Functional characterization of endophytic fungal community associated with *Oryza*
*sativa* L. and *Zea*
*mays* L.. Front. Microbiol..

[54] Di Francesco A (2021). Biocontrol activity and plant growth promotion exerted by *Aureobasidium pullulans* Strains. J. Plant Growth Regul..

[55] Yalage Don SM, Schmidtke LM, Gambetta JM, Steel CC (2021). Volatile organic compounds produced by *Aureobasidium pullulans* induce electrolyte loss and oxidative stress in *Botrytis cinerea* and *Alternaria alternata*. Res. Microbiol..

[56] Hou W, Lian B, Zhang X (2011). CO_2_ mineralization induced by fungal nitrate assimilation. Bioresour. Technol..

[57] Noble AS, Noe S, Clearwater MJ, Lee CK (2020). A core phyllosphere microbiome exists across distant populations of a tree species indigenous to New Zealand. PLoS One.

[58] Ruokolainen L, Ranta E, Kaitala V, Fowler MS (2009). When can we distinguish between neutral and non-neutral processes in community dynamics under ecological drift?. Ecol. Lett..

[59] Zhou J, Ning D (2017). Stochastic community assembly: Does it matter in microbial ecology?. Microbiol. Mol. Biol. Rev..

[60] Louisson Z, Ranjard L, Buckley HL, Case BS, Lear G (2023). Soil bacterial community composition is more stable in kiwifruit orchards relative to phyllosphere communities over time. Environ. Microbiome.

[61] Veach AM, Stegen JC, Brown SP, Dodds WK, Jumpponen A (2016). Spatial and successional dynamics of microbial biofilm communities in a grassland stream ecosystem. Mol. Ecol..

[62] Hugerth LW (2014). DegePrime, a program for degenerate primer design for broad-taxonomic-range PCR in microbial ecology studies. Appl. Environ. Microbiol..

[63] Caporaso JG (2011). Global patterns of 16S rRNA diversity at a depth of millions of sequences per sample. Proc. Natl. Acad. Sci..

[64] Korabecná M, Liska V, Fajfrlík K (2003). Primers ITS1, ITS2 and ITS4 detect the intraspecies variability in the internal transcribed spacers and 5.8S rRNA gene region in clinical isolates of fungi. Folia Microbiol. (Praha).

[65] Dowd SE (2008). Polymicrobial nature of chronic diabetic foot ulcer biofilm infections determined using bacterial tag encoded FLX amplicon pyrosequencing (bTEFAP). PloS One.

[66] Bolyen E (2019). Reproducible, interactive, scalable and extensible microbiome data science using QIIME 2. Nat. Biotechnol..

[67] Callahan BJ (2016). DADA2: High resolution sample inference from Illumina amplicon data. Nat. Methods.

[68] Quast C (2013). The SILVA ribosomal RNA gene database project: Improved data processing and web-based tools. Nucleic Acids Res..

[69] Nilsson RH (2019). The UNITE database for molecular identification of fungi: Handling dark taxa and parallel taxonomic classifications. Nucleic Acids Res..

[70] R Core Team. R: A language and environment for statistical computing. (2022).

[71] Albertsen M, Karst SM, Ziegler AS, Kirkegaard RH, Nielsen PH (2015). Back to basics-the influence of DNA extraction and primer choice on phylogenetic analysis of activated sludge communities. PLoS One.

[72] Oksanen, J. *et al.* Vegan: Community Ecology Package. R package version 2.0-2. (2012).

[73] McMurdie PJ, Holmes S (2013). phyloseq: An R package for reproducible interactive analysis and graphics of microbiome census data. PLOS ONE.

[74] Kembel SW (2010). Picante: R tools for integrating phylogenies and ecology. Bioinformatics.

[75] Goslee, S., Urban, D. & Goslee, M. S. Package ‘ecodist’. *Package ‘ecodist’* (2020).

[76] Liu C, Cui Y, Li X, Yao M (2021). microeco: An R package for data mining in microbial community ecology. FEMS Microbiol. Ecol..

[77] Wickham H, Wickham H (2016). Data analysis. ggplot2.

[78] Love MI, Huber W, Anders S (2014). Moderated estimation of fold change and dispersion for RNA-seq data with DESeq2. Genome Biol..

[79] Põlme S (2020). FungalTraits: A user-friendly traits database of fungi and fungus-like stramenopiles. Fungal Divers..

[80] Gupta NS, Pancost RD (2004). Biomolecular and physical taphonomy of angiosperm leaf during early decay: Implications for fossilization. PALAIOS.

[81] Anesio AM, Abreu PC, Biddanda BA (2003). The role of free and attached microorganisms in the decomposition of estuarine macrophyte detritus. Estuar. Coast. Shelf Sci..

