# Supplementary material for: Oxygen-dependent biofilm dynamics in leaf decay: an in vitro analysis
Source: Sci Rep. 2024 Mar 20;14:6728. doi: 10.1038/s41598-024-57223-7 (PMC10955112; doi:10.1038/s41598-024-57223-7)
Supplement: Supplementary file 1 — Supplementary Information. [file 41598_2024_57223_MOESM1_ESM.docx]

**
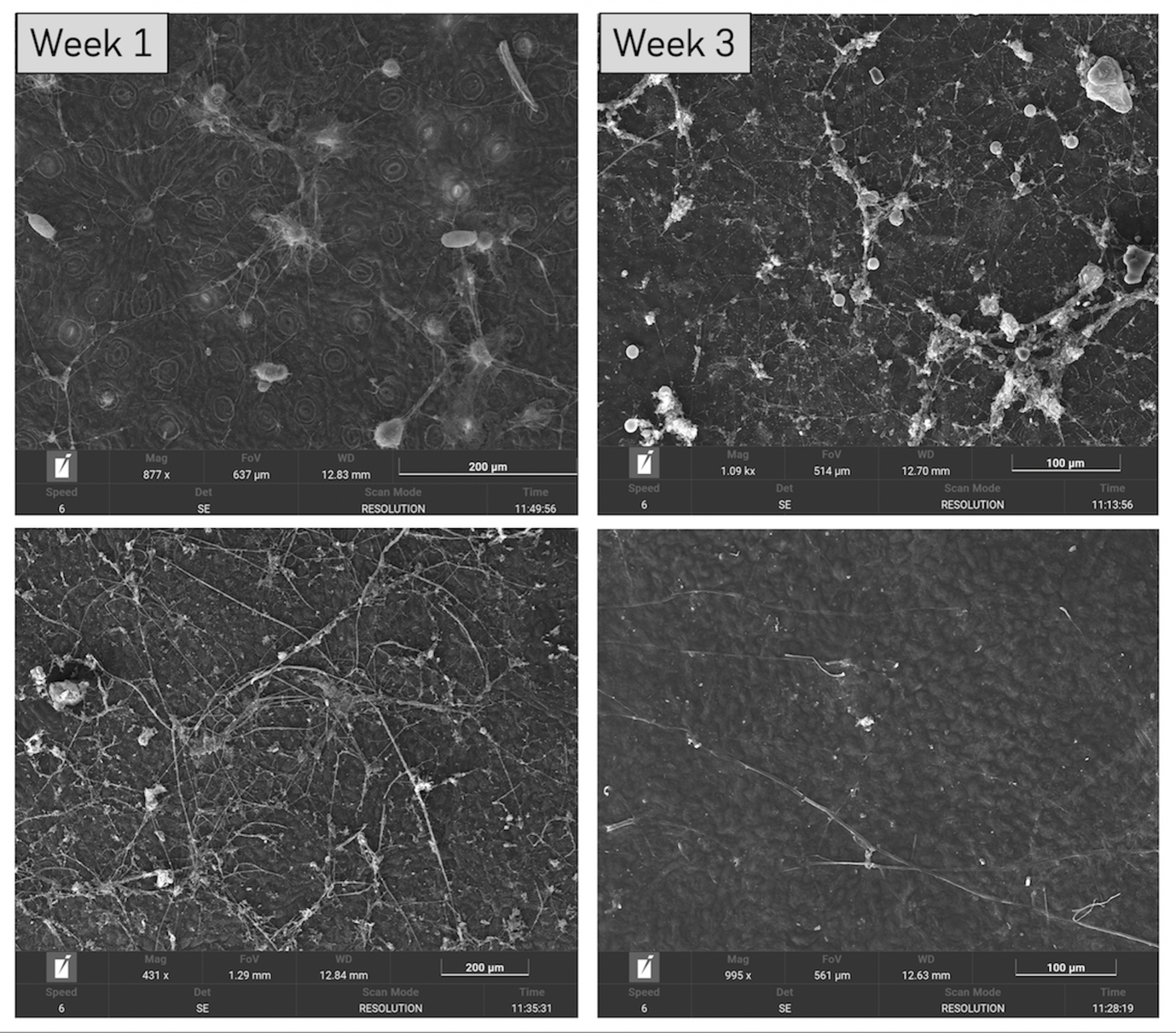
Supplementary Material**

**Figure S1:** SEM images of *Hedera* biofilm sample after 1 week and 3 weeks of experiment under aerobic condition (upper photos) and anaerobic condition (lower photos).


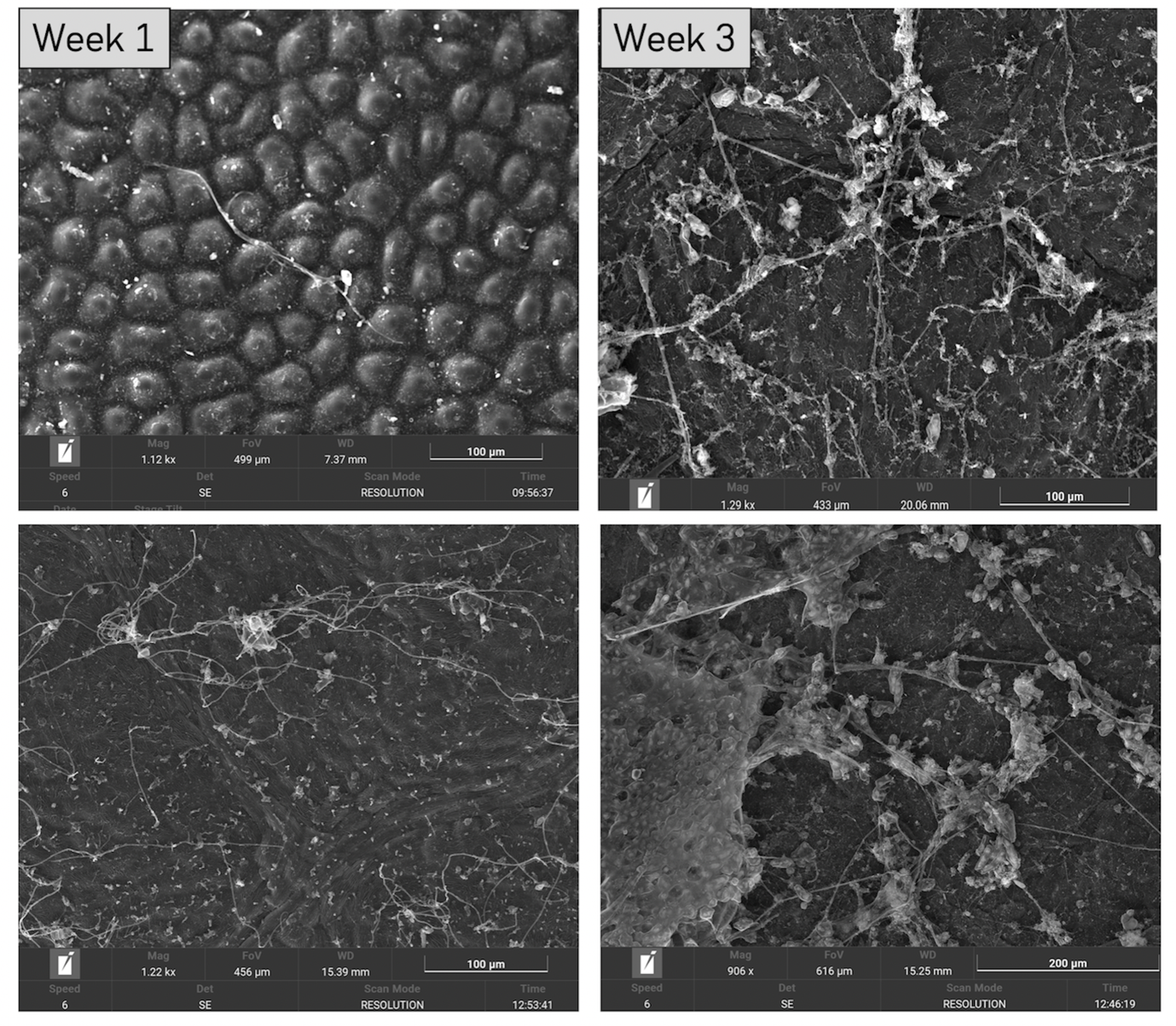


**Figure S2:** SEM images of *Acer* biofilm sample after 1 week and 3 weeks of experiment under aerobic condition (upper photos) and anaerobic condition (lower photos).


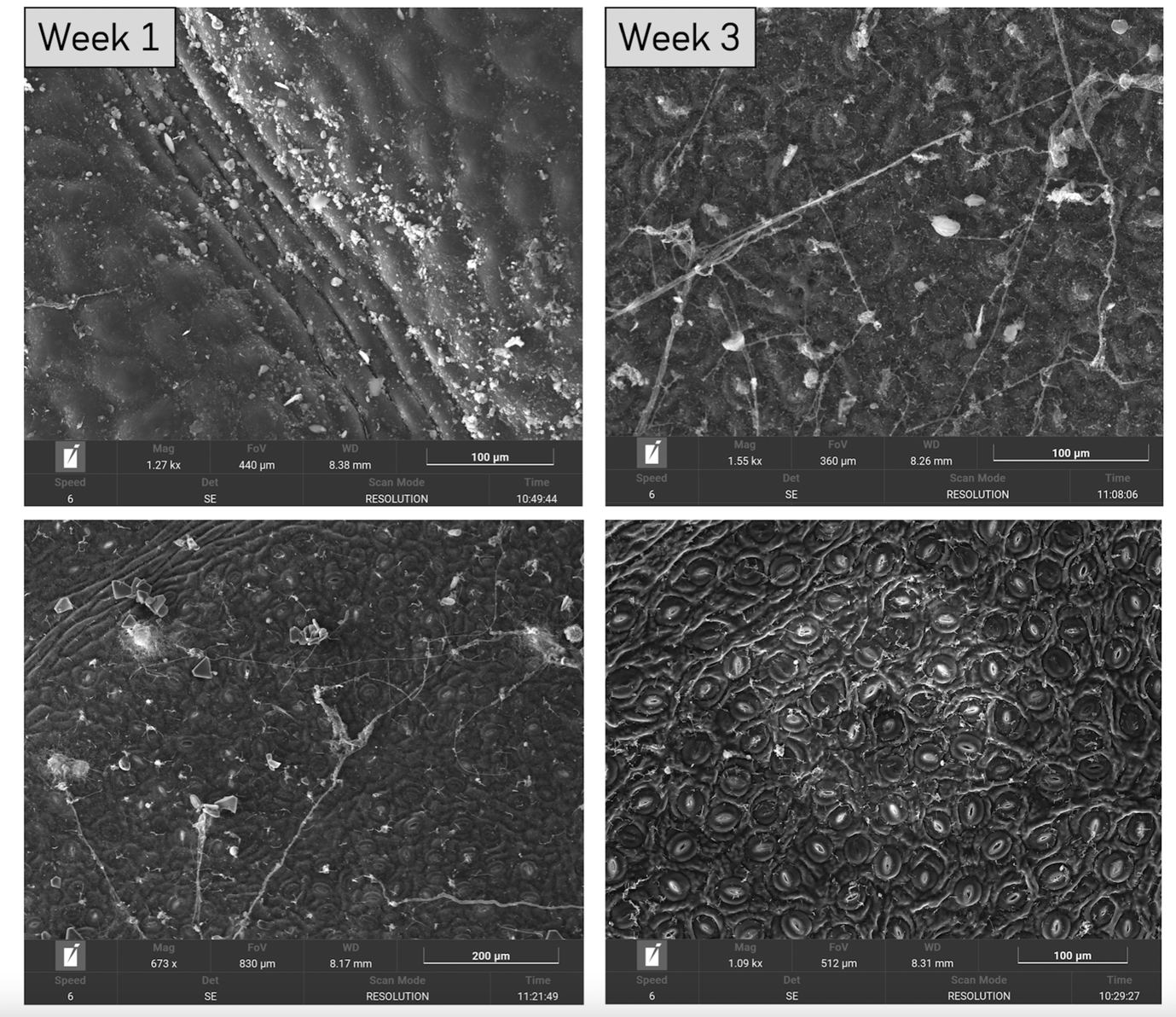


**Figure S3:** SEM images of *Lonicera* biofilm sample after 1 week and 3 weeks of experiment under aerobic condition (upper photos) and anaerobic condition (lower photos).

**
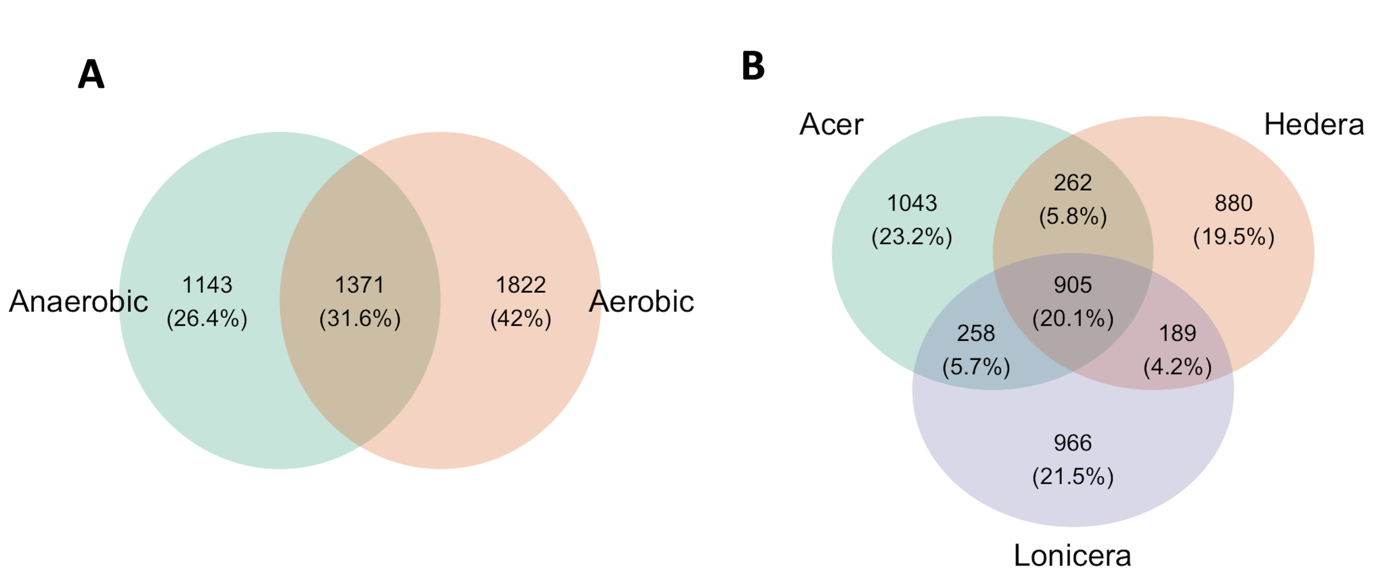
Figure S4: A)** Venn diagrams of the bacterial communities from all samples showing the number of shared ASVs between under aerobic and anaerobic conditions **B)** Venn diagrams of the bacterial communities showing number of shared ASVs between different plant species.


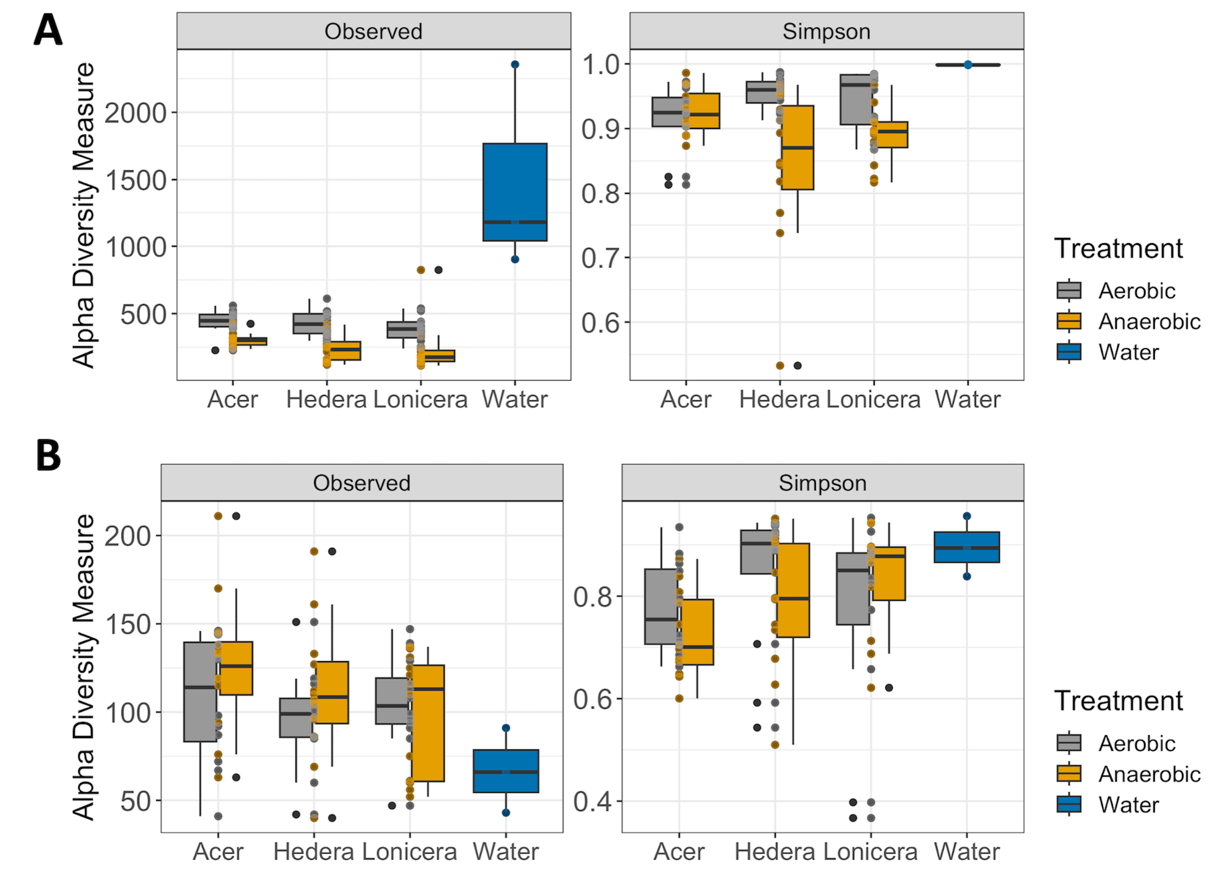


**Figure S5:** Alpha diversity of leaf prokaryotic biofilm communities **(A)** and fungal biofilm communities **(B)** from different plants and water. The boxes show median, 25, and 75 percentiles. The vertical lines show minimum values excluding outliners, and the dots show outliners. The colours represent the oxygen conditions and water.


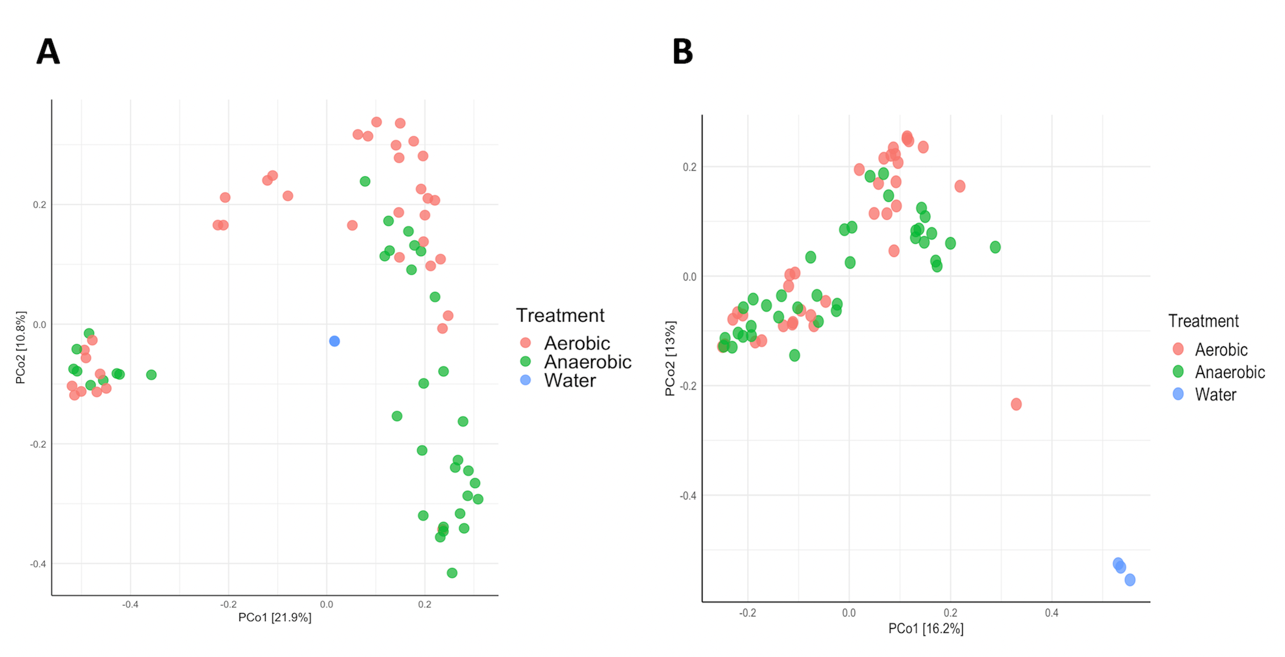


**Figure S6:** Ordination by PCoA of bacterial (A) and fungal (B) lead biofilm communities from aerobic and anaerobic conditions based on Bray-Curtis dissimilarity.


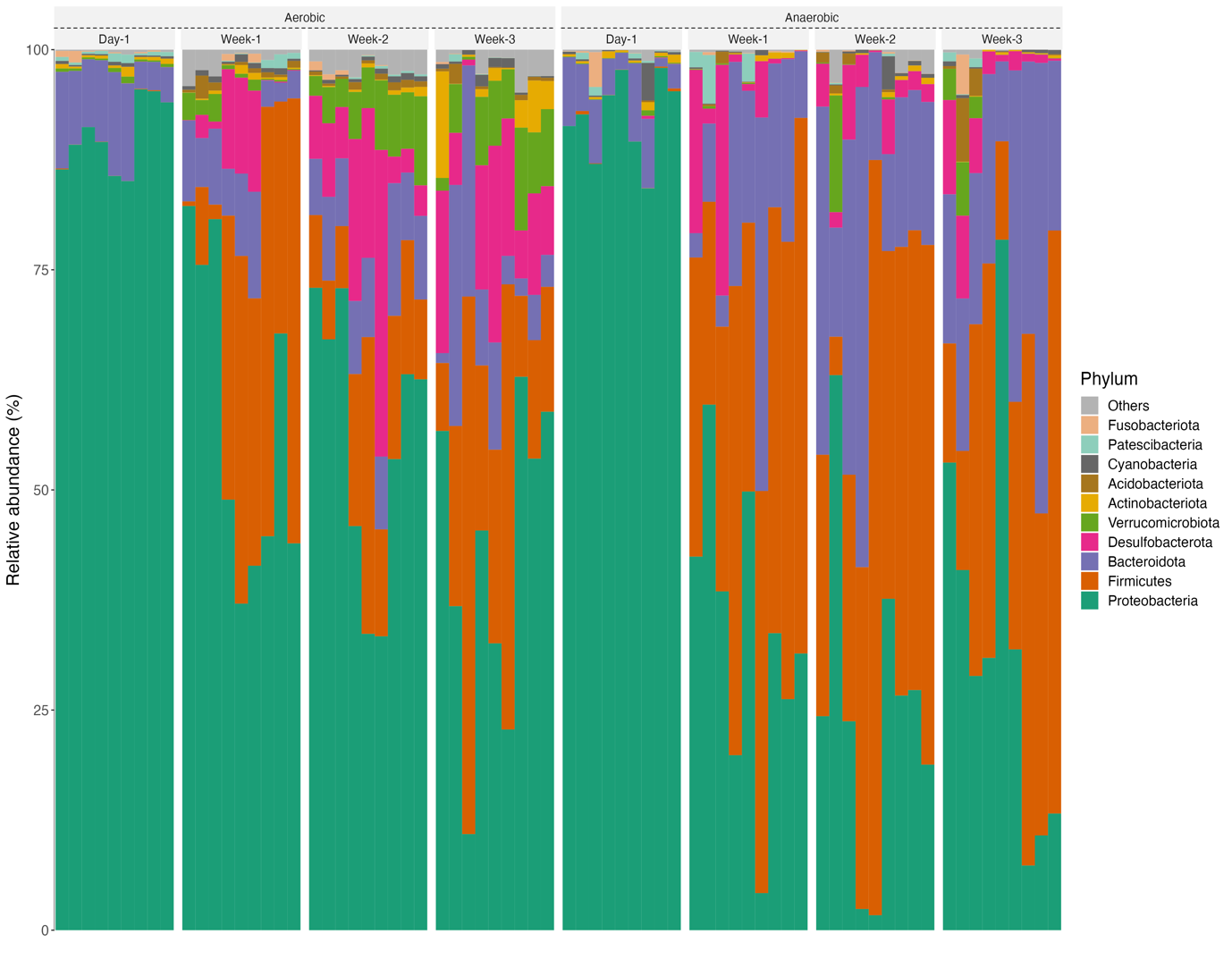


**Figure S7:** Stacked bar plots of the relative abundance of the abundant prokaryotic phyla over 3 weeks of experiment (representing >1% of the total relative abundance).


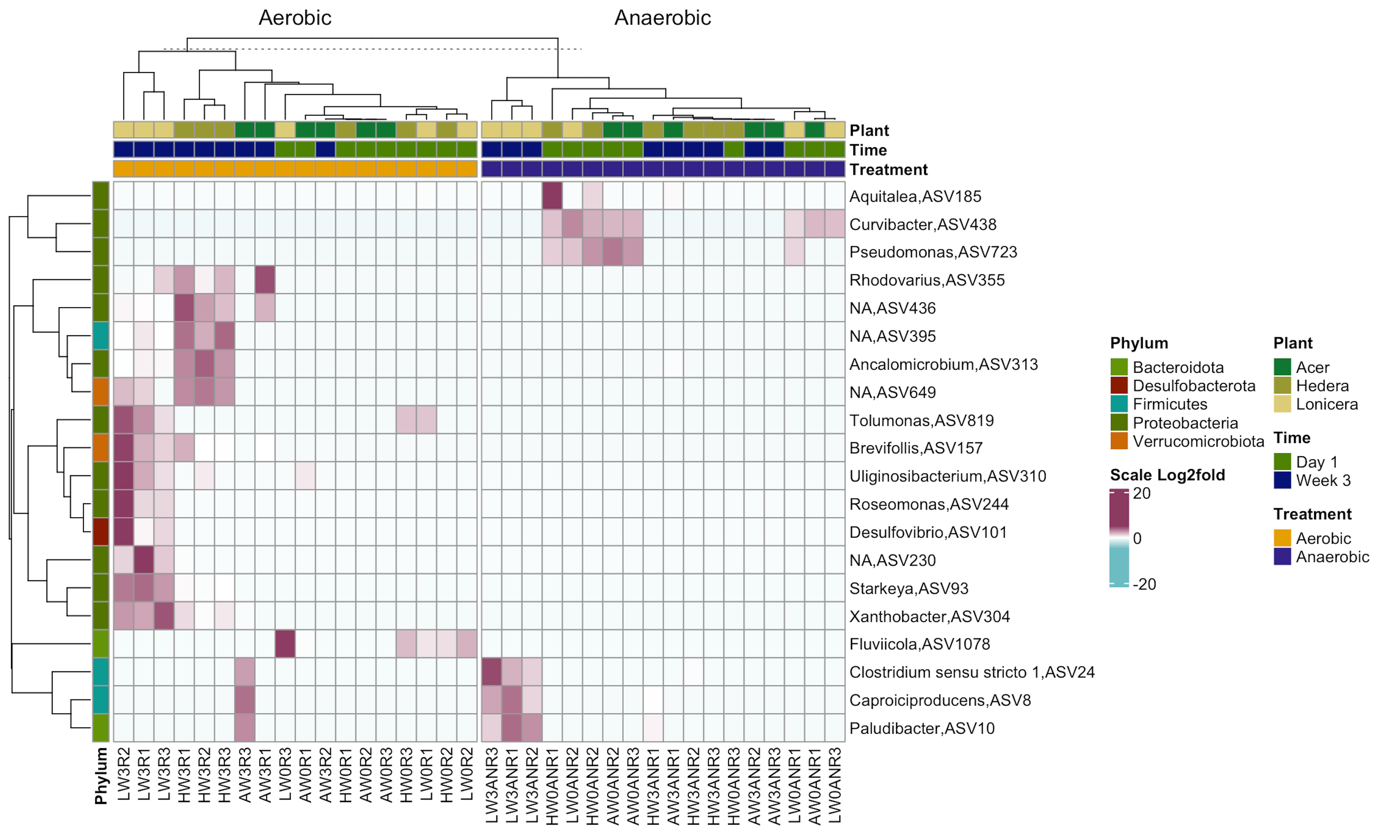
**Figure S8**: Heatmap showing significantly different amplicon sequence variants (ASVs) between leaf prokaryotic biofilms samples from aerobic and anaerobic conditions, based on DESeq2 results (p_adj._ < 0.01). Samples have been grouped initially by treatment (aerobic, anaerobic), then by time (day 1, week 3) and by plant genus (*Acer*, *Hedera*, *Lonicera*). ASVs are labelled according to phylum level taxonomy indicated on the left. The shaded cells represent the relative abundance of each ASV on a Log2fold scale with dark red indicating higher abundance and blue indicating lower abundance.


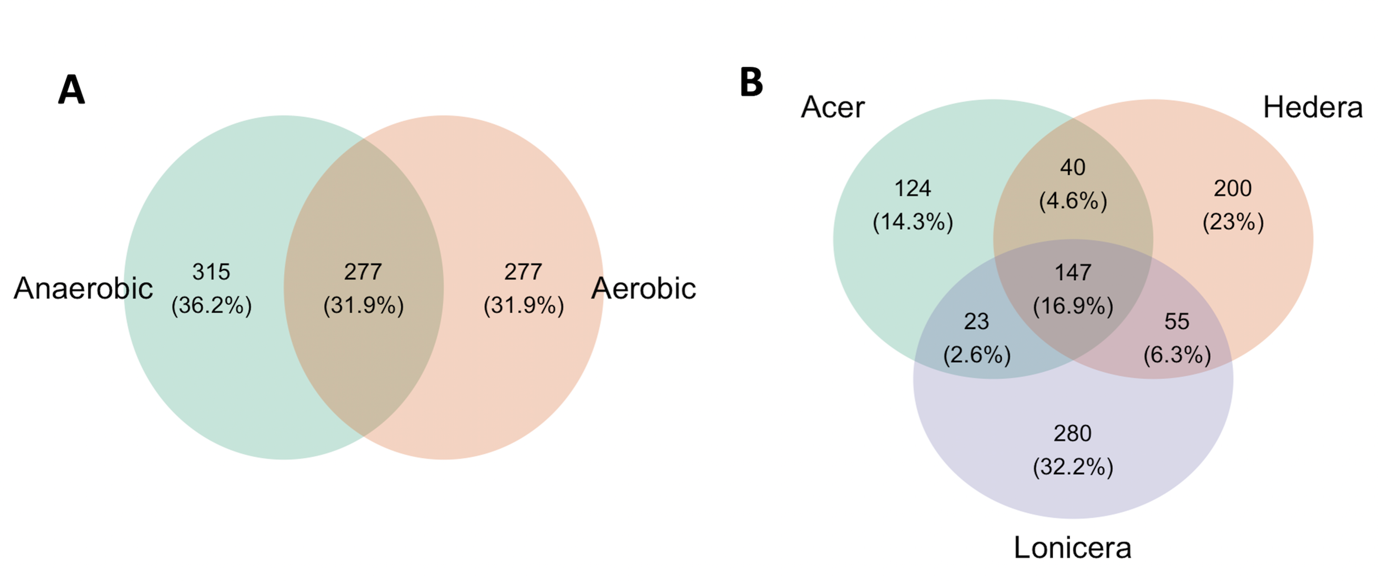


**Figure S9: A)** Venn diagrams of the fungal communities from all samples showing number of shared ASVs between under aerobic and anaerobic conditions **B)** Venn diagrams of the fungal communities showing number of shared ASVs between different plant species.


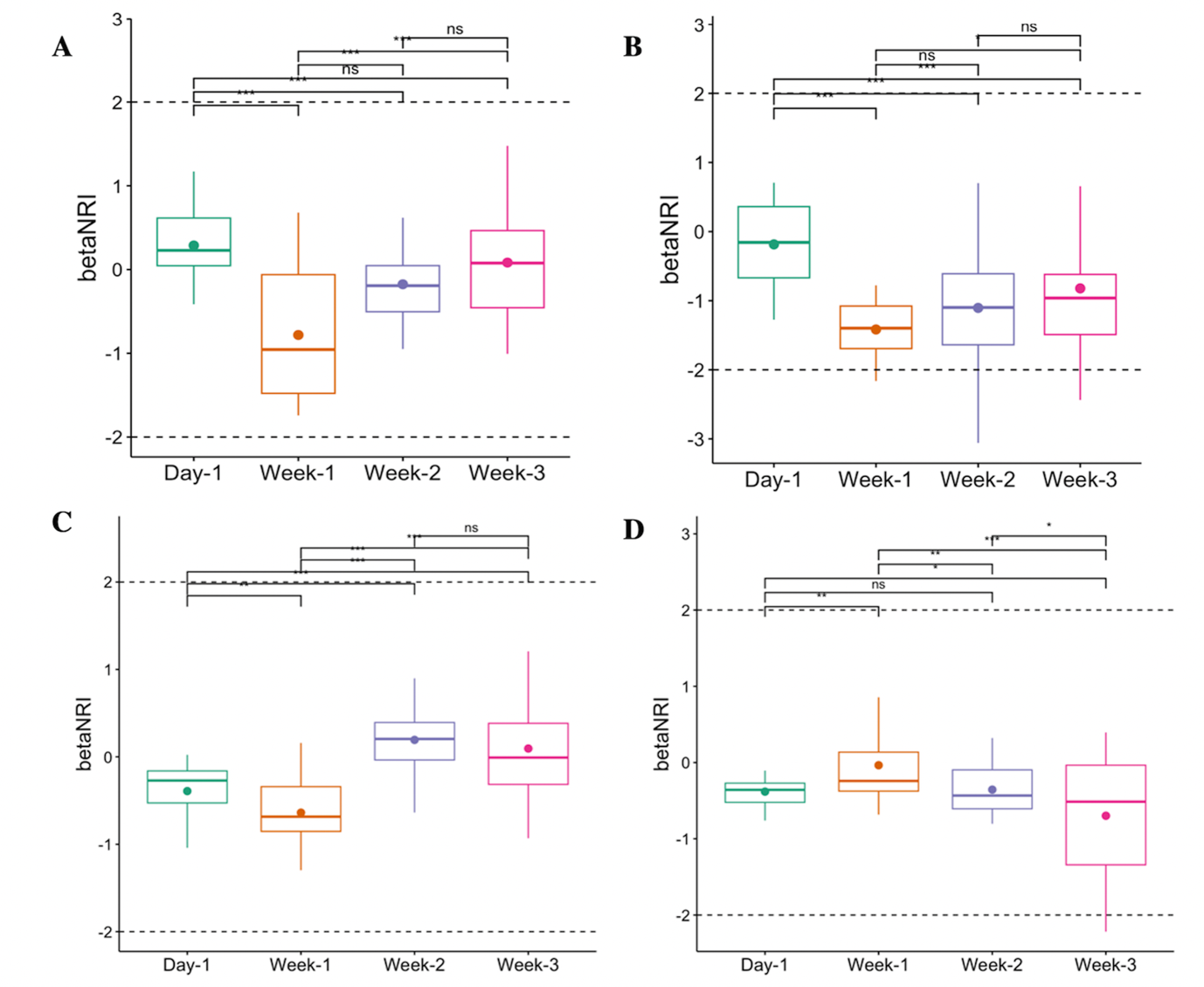
**Figure S10:** betaNet relatedness index (betaNRI) over time for bacterial biofilm communities (A- aerobic condition, B- anaerobic assay) and fungal biofilms (C- aerobic condition, D – anaerobic assay). BetaNRI <0 represents phylogenetic divergence, while betaNRI >0 indicates phylogenetic convergence (p_adj_ < 0.05).


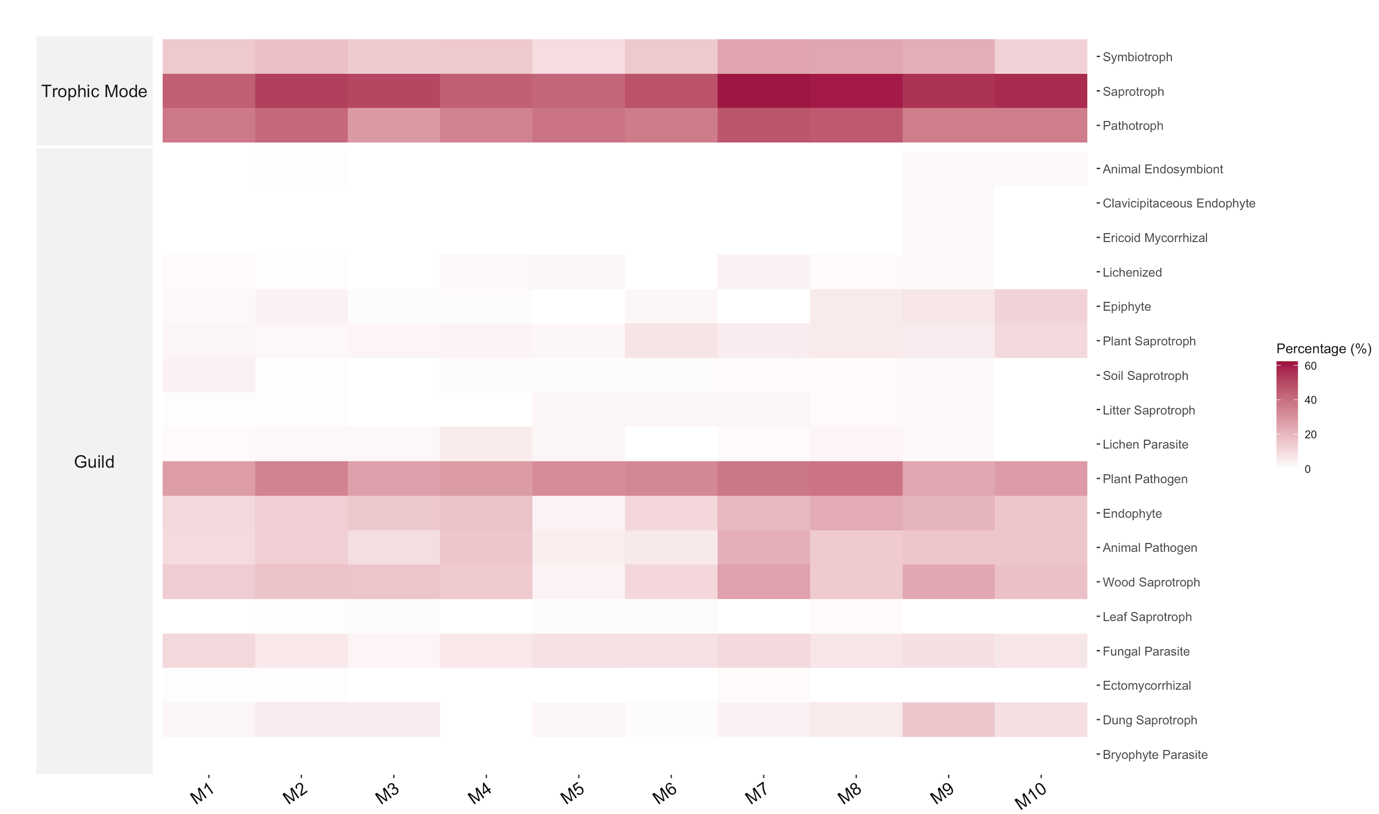


**Figure S11:** Functional structure (based on FAPROTAX analysis) of the prokaryotic biofilm communities from all plant samples.


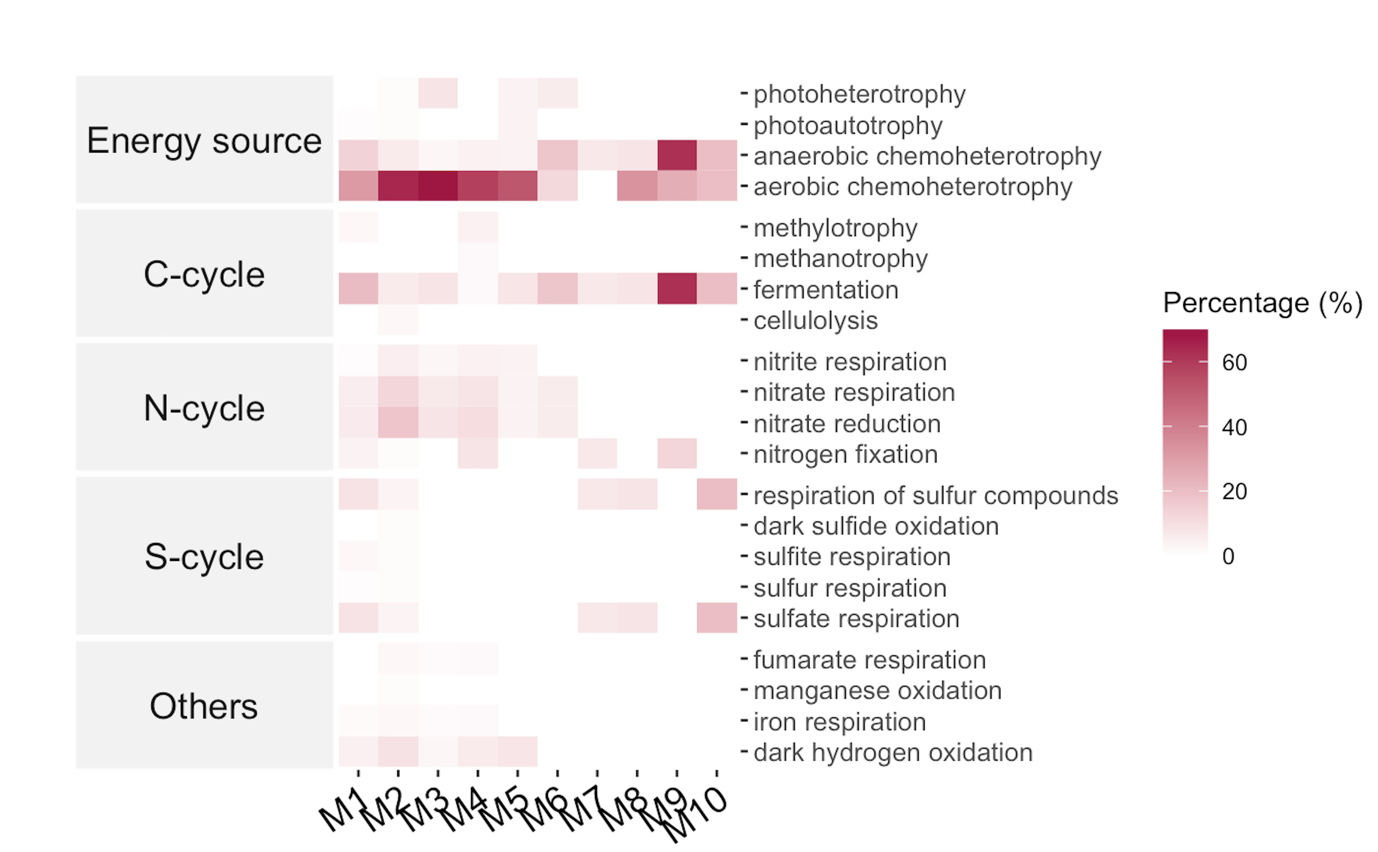


**Figure S12**: Classified fungal trophic modes by FUNGuild.

**Table S1:** Results from PERMANOVAs to determine differences in the biofilm community composition. Values in bold indicate a significant p-value (P < 0.05).

|  | 16S | | | ITS | | |
| --- | --- | --- | --- | --- | --- | --- |
| Variable | **R2** | **F** | **P-Value** | **R2** | **F** | **P-Value** |
| Plant (*Hedera, Acer, Lonicera*) | 0.032 | 1.158 | 0.235 | 0.714 | 0.032 | 0.254 |
| Week (Day 1- Week 3) | 0.130 | 3.3895 | **0.001** | 0.170 | 4.6528 | **0.001** |
| TreatmEnt (Aerobic-Anaerobic) | 0.027 | 1.991 | **0.021** | 0.025 | 1.843 | 0.05 |

**Table S2:** Estimated normalized stochasticity ratio based on the NST package.

1. Bacterial biofilm community

| **Time** | **ST.i.bray** | **NST.i.bray** | **MST.i.bray** | **SES.i.bray** |
| --- | --- | --- | --- | --- |
| Day 1 | 0.7901708 | 0.7401623 | 0.6232623 | -1.344824 |
| Week 1 | 0.6467311 | 0.3666158 | 0.3104136 | 3.252523 |
| Week 2 | 0.6281169 | 0.2745662 | 0.2355986 | 3.995697 |
| Week 3 | 0.6857876 | 0.2841127 | 0.2206236 | 3.249257 |

1. Fungal biofilm community

| **Time** | **ST.i.bray** | **NST.i.bray** | **MST.i.bray** | **SES.i.bray** |
| --- | --- | --- | --- | --- |
| Day 1 | 0.6978843 | 0.5696998 | 0.4451246 | 0.9964417 |
| Week 1 | 0.6607544 | 0.5245228 | 0.4152851 | 1.4586824 |
| Week 2 | 0.5470213 | 0.339752 | 0.2914393 | 2.5231662 |
| Week 3 | 0.6955899 | 0.5341715 | 0.3318168 | 1.4640793 |
